# A Stage-Based Approach to Therapy in Parkinson’s Disease

**DOI:** 10.3390/biom9080388

**Published:** 2019-08-20

**Authors:** Claudia Carrarini, Mirella Russo, Fedele Dono, Martina Di Pietro, Marianna G. Rispoli, Vincenzo Di Stefano, Laura Ferri, Filomena Barbone, Michela Vitale, Astrid Thomas, Stefano Luca Sensi, Marco Onofrj, Laura Bonanni

**Affiliations:** Department of Neuroscience, Imaging and Clinical Sciences, University G. d’Annunzio of Chieti-Pescara, 66100 Chieti, Italy

**Keywords:** Parkinson’s disease, motor symptoms, non-motor symptoms, non-pharmacological therapy, l-dopa, dopamine-agonists, acetylcholinesterase inhibitors, monoamine oxidase inhibitors, amantadine, anticholinergics

## Abstract

Parkinson’s disease (PD) is a neurodegenerative disorder that features progressive, disabling motor symptoms, such as bradykinesia, rigidity, and resting tremor. Nevertheless, some non-motor symptoms, including depression, REM sleep behavior disorder, and olfactive impairment, are even earlier features of PD. At later stages, apathy, impulse control disorder, neuropsychiatric disturbances, and cognitive impairment can present, and they often become a heavy burden for both patients and caregivers. Indeed, PD increasingly compromises activities of daily life, even though a high variability in clinical presentation can be observed among people affected. Nowadays, symptomatic drugs and non-pharmaceutical treatments represent the best therapeutic options to improve quality of life in PD patients. The aim of the present review is to provide a practical, stage-based guide to pharmacological management of both motor and non-motor symptoms of PD. Furthermore, warning about drug side effects, contraindications, as well as dosage and methods of administration, are highlighted here, to help the physician in yielding the best therapeutic strategies for each symptom and condition in patients with PD.

## 1. Introduction

Parkinson’s Disease (PD) is a neurodegenerative disorder characterized by motor and non-motor features, due to a progressive loss of dopaminergic neurotransmission in basal ganglia [1]. Decrease of dopamine-containing neurons within substantia nigra and presence of Lewy bodies inclusions are the core pathological findings in PD [2].

According to Movement Disorder Society-PD (MDS-PD) criteria [3], the clinical diagnosis of PD has centered on a defined motor syndrome (Parkinsonism), based on three cardinal motor symptoms (MS), such as bradykinesia, rigidity, and resting tremor. However, non-motor symptoms (NMS), which include insomnia [4], depression, anxiety [5], cognitive decline [6], apathy [7], neuropsychiatric disturbances [8], and autonomic dysfunctions [9], can be present at the onset and during disease progression. Therefore, PD increasingly compromises quality of life and activities of daily living, although a high variability in clinical presentation and in disease progression can be observed among people affected [10]. Thus, the Hoehn and Yahr scale is commonly used to compare groups of patients and to provide an indicative assessment of disease progression, ranging from stage I (only unilateral involvement) to stage V (wheelchair bound or bedding) [11]. In an early stage, symptoms are usually mild and unilateral with a complete response to treatment. Although symptoms tend to progress and motor symptoms affect the contralateral side, at first drug response is commonly effective. During the course of the disease, treatment response decreases, and anti-parkinsonian drugs can potentially induce side effects. After a prolonged disease duration, patients can develop several NMS for which current treatments are limited [12]. Previous studies have reported that the mean duration of disease ranges from 6.9 to 14.3 years and the onset of dementia is the highest predictor of increased mortality [13].

In this review both clinical features of PD, motor and non-motor, are described in association with disease progression (Table 1). Our aim is to provide an up-to-date review of pharmacological treatments during different stages of the disease. 

## 2. Early Stage

Several non-motor symptoms and signs of PD can be observed in the very early stages of disease [14]. According to staging system of Braak, pathological process of PD may not begin in substantia nigra pars compacta [15]. Indeed, α-synuclein deposition seems to firstly involve the anterior olfactory nucleus and dorsal motor nucleus of the vagus. A further subsequent finding suggests that peripheral autonomic ganglia and unmyelinated lamina-1 spinal cord neurons may also be involved in the initial pathological phases [16]. These findings are consistent with PD typical early non-motor features, such as olfactory dysfunction, rapid eye movement (REM) sleep behavior disorder (RBD), constipation, anxiety, and depression [17].

The quantitative assessment of clinical symptoms and progression of the course is an essential component of any therapeutic trial in PD (Table 2) [1]. Nowadays, many types of medication are available for symptomatic treatment of early stages of PD [18,19]. In an early stage, timing and therapeutic choice depend on patient and disease characteristics. Due to PD variability, it is known that there is not a single preferred therapy to use. The most relevant patient-related factors are age, which has important implications in tolerability of certain drug classes, and severity of symptoms, because antiparkinsonian drug efficacy tends to vary into different classes of pharmacological agents. Thus, symptom severity categorization should be based on a holistic evaluation, including neurologic examination and a detailed assessment of how symptoms affect daily functions and quality of life [20]. 

### 2.1. Treatments for Non-Motor Symptoms in the Early Stage

#### 2.1.1. Olfactory Dysfunction

Olfactory dysfunction is a typical symptom complained about in the early stage of the disease. It seems to affect more than 80% of patients, but only about 40% of them are aware of an impaired sense of smell [21]. A recent study has reported that olfactory dysfunction is associated with decreased dopamine transporter binding in more than 40% of patients [22]. In a small cohort of patients with idiopathic RBD (iRBD), the presence of smell impairment has been detected as a predictive value for conversion to PD and to Parkinson disease with dementia (PDD) over 5 years [23].

According to previous studies, hyposmia seems to be related to neuronal loss in cortico-medial amygdala or to decreased dopaminergic neurons in the olfactory bulb. This disturbance, which generally affects both nostrils, tends to correlate directly with disease duration and severity [24,25]. Furthermore, smell loss has been related to an increased risk of cognitive decline and it is consequently a prodromal symptom of PDD onset. Currently, hyposmia cannot be treated by any anti-PD drugs [26,27].

#### 2.1.2. REM Sleep Behavior Disorder (RBD)

RBD is a parasomnia characterized by loss of REM sleep atonia, which results in vigorous, violent motor jerks, and nocturnal vocalizations in association with vivid dreams [28]. The prevalence of iRBD is not well known, although about 5%–8% of the Caucasian population aged over 60 years seems to be affected by this sleep disturbance [29]. RBD occurs in up to 50% of PD patients and it can precede by several years the onset of synucleinopathies, such as PD, Dementia with Lewy bodies (DLB), and Multiple system atrophy (MSA) [30]. Some evidence suggests that RBD, which is a potential predictor of disease severity, can be associated with an akinetic-rigid subtype of PD, characterized by a more pronounced autonomic dysfunction and an increased risk of cognitive impairment or dementia [31]. Diagnosis is based on polysomnography (PSG), because other pathological conditions, such as non-REM parasomnias and obstructive sleep apnea, can mimic RBD [17].

Clonazepam and melatonin are first-line treatments for RBD. Clonazepam is a long-lasting benzodiazepine, which does not suppress motor tone during REM sleep but prevents dream enactment behavior onset, through uncertain mechanisms [25]. The treatment dose is 0.5 to 2.0 mg before bedtime and it is rarely associated with dosage tolerance and side effects (<10% of cases), such as daytime sedation, worsening of obstructive and central sleep apnea, alopecia, depression, memory impairment, and gastroesophageal reflux. Thus, Clonazepam is completely or partially successful in up to 90% of patients with RBD [32,33]. Although it is the most common used drug for RBD, current evidence about its effectiveness have only been based on observational studies (retrospective cohorts and case-series). In addition, clinical studies have been conducted with small cohorts of patients and without using PSG to value treatment response [34].

Melatonin can be prescribed as a first-line therapy for patients with poor tolerance or contraindications to Clonazepam, such as dementia, obstructive sleep apnea, and an increased risk of falls [35]. It is a hormone released by the pineal gland in a circadian pattern, whose levels rise shortly after nightfall reaching a peak in the middle of the night [36]. Thus, its role is to regulate the sleep-wake-cycle. Treatment should be administered at high doses (2 to 10 g) at bedtime. Melatonin can be used in monotherapy or as add-on therapy in PD patients with RBD [37,38], as suggested in previous studies in which doses of this substance have successfully treated RBD with few side effects [39,40].

A limited number of studies have examined the efficacy of melatoninergic agonists in RBD, such as Agomelatine, Ralmeteon, and Tasimelton. One case series [41] has reported a positive effect of agomelatine in three patients with iRBD without adverse effects. Recently, two studies [39,42] have reported the clinical effectiveness of Ralmeteon, a new melatoninergic agonist, already released in some countries to treat insomnia. To date, no studies on Tasimelton efficacy for RBD treatment have been published.

Dopaminergic drugs have demonstrated contrasting profiles of efficacy in RBD. A prospective case series has reported RBD onset after 1 year of Levodopa (l-dopa) treatment in five out of 15 PD patients [43]. Another study has reported that PD patients with RBD generally used higher doses of l-dopa [44] in comparison with those without RBD at the same stage of disease. Pramipexole, in doses up to 2–4 mg at bedtime, has been shown to be effective in 62%–89% of patients with iRBD, RBD associated with mild cognitive impairment, or RBD with mild PD [45,46]. In contrast, another study has not reported severity or frequency decrease of RBD when pramipexole has been added to a stable l-dopa dose [47].

Acetylcholinesterase inhibitors (AChEI), such as Donepezil, in doses of 10–20 mg, and Rivastigmine, in doses up to 6 mg at bedtime, seem to be effective in RBD treatment. The role of AChEI in RBD is supported by experimental studies which have documented that cholinergic neurons, situated in upper pontine tegmentum and mesencephalon, become active during REM phase and may regulate REM sleep and atonia [48,49]. These neurons are involved via direct or indirect inhibitory descending pathway through the reticular magnocellular tract and glycinergic neurons on the spinal motor neurons [50]. Thus, neurodegenerative processes in PD may induce a dysfunction of this nucleus and its afferent or efferent pathways, resulting in loss of the normal inhibition of motor neurons [51].

Effects of Memantine on sleep disturbances have been evaluated in a multicenter study, in which [45] patients with RBD were included. Memantine, a glutamatergic antagonist, might reduce these disorders, but its validity has been limited by the absence of PSG to support RBD diagnosis [52]. Sodium oxybate, an agent used to treat narcolepsy, has been documented to be effective as monotherapy or as add-on therapy in RBD in some cases [53,54]. The role of Cannabidiol (CBD) in PD with RBD has also been evaluated in a pilot study. CBD, used in doses ranging from 75 to 300 mg for six consecutive weeks, has shown up to 80% frequency decrease of sleep disturbances [55].

#### 2.1.3. Constipation

Constipation, the most common gastrointestinal disturbance in PD, may occur in up to 29% of patients [56] and it can be present 20 years before the onset of motor symptoms [57]. Therefore, disease-related pathophysiological mechanisms and drug side effects have been identified to cause this gastrointestinal disturbance, although physical weakness and lifestyle risks, such as reduced fluid intake, may promote its onset [58]. According to recent evidence, prebiotic fibers and probiotics have been considered efficacious and clinically useful to treat constipation [59]. Macrogol, an osmotic laxative, is an alternative drug with a good efficacy and safety profile. Lubiprostone, an intestinal chloride secretagogue, is an oral bicyclic fatty acid derived from prostaglandin E1 that selectively activates type 2 chloride channels (ClC-2) in the apical membrane of the gastrointestinal epithelium. It therefore softens stools and increases motility [60]. Currently, its use is not accepted in all countries. Use of antiparkinsonian drugs for constipation remains a subject of controversial debate. Whereas some authors have related constipation to dopaminergic treatments [60,61], others have contrarily suggested that l-dopa might improve gastrointestinal symptoms [62]. Moreover, anticholinergics drugs are contraindicated in PD patients suffering from constipation [63].

#### 2.1.4. Depression and Anxiety

Depression may precede motor symptoms in 30% of PD patients [64]. It may be explained through a decreased activity in orbitofrontal and limbic cortices. Moreover, previous studies have documented a correlation between disease severity and depression [65]. The prevalence of anxiety in PD patients is 25%–40%, which is generally characterized by panic attacks, phobias, and generalized anxiety disorder [66]. Approximately 92% of patients tends to manifest anxiety disturbances in association with depression. It is suggested that it can be related to several neurotransmitter deficits, involving serotonergic, adrenergic, and dopaminergic systems.

According to a recent update [60], Tricyclic Antidepressants (TCAs) can be considered “possibly useful” to treat depression. Evidence seems to be insufficient for the use of Amitriptyline, although a recent review has shown that it seems to be more effective than other antidepressants [67]. Treatment of PD patients with TCAs may result in psychosis, sedation, and daytime sleepiness as well as in cognitive dysfunction or delirium when used in patients with PDD. Regarding the use of Selective Serotonin Reuptake Inhibitors (SSRIs) and Selective Serotonin-Norepinephrine Reuptake Inhibitors (SNRIs), Venlafaxine seems to present the best effective profile, although other antidepressants such as Citalopram, Sertraline, Paroxetine, and Fluoxetine are considered possibly useful in clinical practice. As a result of conflicting efficacy data, there are still “insufficient evidence” for all SSRIs reviewed. SSRIs have been found to present an improved safety profile in comparison with TCAs in studies conducted in psychiatric populations. SSRIs may worsen PD tremors in up to 5% of patients and occasionally worsen other features of parkinsonism [68]. In addition, Citalopram, used in a daily dose of more than 20 mg, may cause a QT interval (QTc) prolongation in patients over 60 years [60]. Moreover, Serotonin syndrome may occur when SSRIs or SNRIs are used in association with Monoamine oxidase B inhibitors (MAO-BIs), such as Selegiline and Rasagiline [69]. PD-related depression may also be treated with second generation non-ergot Dopamine agonists (DA) such as Pramipexole [70]. A new study has evaluated Rotigotine with negative outcomes. Currently, insufficient evidence is present for the use of MAO-BIs [60].

Anxiety disturbances in PD patients may contribute to relevant impairments in cognitive functions, in motor performances, and in quality of life. Pharmacological agents, including benzodiazepines, Buspirone, and SSRIs, may reduce anxiety. For example, although benzodiazepines (such as Bromazepam) seem to be effective, their long-term use is associated with confusion, gait disturbance, and increased risk of falls. However, because of the current lack of evidence in efficacy and safety profile, pharmacological therapies should be used under careful evaluation [70].

#### 2.1.5. Impulse Control Disorder

Impulse Control Disorder (ICD) (i.e., hypersexuality, gambling, binge-eating, compulsive shopping or hobbying, punding, and homeostatic hedonism disorders) are more frequent in young, male patients [71]. ICD main risk factors include dopaminergic replacement therapy, in particular with DAs (14%–17% of cases), and disease duration [72,73]. In these patients, DA therapy should be discontinued (or at least reduced until ICD cessation) with proportional l-dopa dose adjustment. However, some patients could experience DAWS, with mood, autonomic, and sleep disorders, requiring careful symptomatic management [72].

Other NMS, which can appear in the early stage but are prominent during the progression of disease, will be later discussed.

### 2.2. Treatments for Motor Symptoms in the Early Stage

#### 2.2.1. l-Dopa

The most effective treatment of PD still remains l-dopa. The global antiparkinsonian efficacy of l-dopa is so dramatic and predictable that a positive therapeutic response is used to define the disease itself. The major issue regarding introducing l-dopa is when to start treatment, considering the well-known effects of long-term use [74]. It has been largely reported that the premature therapeutic introduction of l-dopa, especially in young patients, leads to develop long-term side effects such as dyskinesias and motor fluctuations. For this reason, some authors have recommended the l-dopa-sparing strategy, which consists of the use of other antiparkinsonian medication in young PD who are more likely to develop dyskinesias and fluctuations related to long term use of l-dopa. The reason why l-dopa long term use determines the onset of motor dysfunction might be due to the toxicity of l-dopa upon dopaminergic neurons. In an in vitro experimental setting, it has been demonstrated that l-dopa metabolites increase oxidative stress and they are toxic to cultures of mesencephalic neurons [75]. However, there is no in vivo evidence that l-dopa accelerates disease progression and, as it was shown by an ELLDOPA (Early versus Later Levodopa therapy in PD patients) study [76], that patients in l-dopa treatment actually are less impaired even after stopping medication for a few weeks compared to patients who delay l-dopa therapy. Previous studies have also documented that dopaminergic depletion is involved in visual dysfunction in PD patients, therefore l-dopa therapy may improve visual disturbances investigated by electrophysiological tests [77,78].

l-dopa formulations are generally associated with a peripheral Dopa decarboxylase inhibitors (more frequently carbidopa or benserazide) in order to inhibit peripheral metabolism of l-dopa. Slight differences in terms of tolerability are associated with the treatment with l-dopa + benserazide (Madopar) compared to l-dopa + carbidopa (Sinemet). In a triple-blind trial [79], patients were allocated at random to treatment with either l-dopa + benserazide ratio 4:1 (Madopar) or l-dopa + carbidopa ratio 10:1 (Sinemet) using dosage schedules recommended by the manufacturers which they had to adhere to for 6 months. Results showed that the effect of the two schedules on the parkinsonian symptoms were equal and they appeared equally fast but the frequency of gastrointestinal side-effects and involuntary movements seemed higher and more severe for Sinemet than for the Madopar group.

Recently, oxidative stress (OS) has been identified as one of the major factors involved in dopaminergic degeneration. Several treatments based on antioxidant therapies, such as vitamin E, creatine, coenzyme Q10, and mitoquinone, have been demonstrated effective in animal models of PD [80]. Several authors have explored the possibility to reduce OS. For example, 1-*O*-Hexyl-2,3,5-trimethylhydroquinone (HTHQ) is a potent antioxidative agent, studied in vitro by Jin Park et al., which seems to be involved in inhibition of l-dopa-induced cytotoxicity because of its role in modulating reactive oxygen species formation in PC12 cells [81]. Moreover, Nikolova et al. [82] developed an experimental model using healthy mice and some OS indicators, such as malondialdehyde, protein carbonyl content and advanced glycation end products, were evaluated in blood plasma. In this study, healthy mice were divided into four groups: the control group, the group treated only with l-dopa, and the remaining two groups, which were respectively pre-treated with Ascorbic acid (400 mg/kg) and Rose Oil (400 mg/kg), two antioxidants. A relevant increase of OS indicators levels in l-dopa treated mice compared to controls has been shown, whereas the same parameters have been decreased in both groups pre-treated with anti-oxidant compared to the same controls [82]. These evidences suggest that antioxidants may have a relevant role in protecting dopaminergic neurons from l-dopa induced cytotoxicity [83].

#### 2.2.2. Dopamine Agonists

DAs are commonly divided into two groups: ergot and non-ergot-derived agonists. It is noteworthy to consider that DAs are ineffective in patients without therapeutic responses to l-dopa, but they might have a role in patients with advanced PD as a treatment for motor complications related to l-dopa [84]. The use of DAs in advanced PD is discussed separately. Neurologists have debated for years whether to select l-dopa or DAs as initial therapy in relatively young PD patients. DAs should be generally avoided in patients over 70 years due to poor tolerability. Furthermore, presence of cognitive dysfunction at baseline may also influence susceptibility to side effects [82].

#### 2.2.3. Non-Ergot Dopamine Agonists

Three Non-Ergot DAs, such as Pramipexole, Ropinirole, and transdermal Rotigotine, are commonly used in PD and they have been shown to be effective as monotherapy in patients in an early stage of the disease [85,86]. Few studies have compared efficacy between various DAs and no significant difference has been found [87]. Pramipexole immediate release is usually started at 0.125 mg three times daily. Its dose should be increased gradually by 0.125 mg per dose every 5–7 days. Pramipexole extended release is usually started at 0.375 mg daily at bedtime, eventually increased by 0.375 mg every 5–7 days. Commonly, the mean daily dose used is 1.5–4.5 mg. Dose adjustments are required for renal insufficiency, but the extended-release formulation is not recommended in patients with a creatinine clearance <30 mL/minute [88]. Ropinirole immediate release is usually started at 0.25 mg three times daily and it should be increased gradually by 0.25 mg per dose each week for 4 weeks to a total daily dose of 3 mg. After 4 weeks, its dose may be increased weekly by 1.5 mg a day up to a maximum total daily dose of 24 mg. The extended release formulation is usually started at 2 mg daily at bedtime and increased by 2 mg every 5–7 days, up to a maximum of 24 mg. Benefits most commonly occur in a dose range of 12–16 mg daily. Transdermal Rotigotine is a once-daily patch which is usually started at 2 mg/daily and titrated weekly by increasing the patch size in 2 mg/daily increments to a dose of 6 mg/daily. Transdermal formulations are generally preferred in order to avoid issues such as renal impairment or inability to swallow whole pills.

Adverse effects caused by DAs are similar to those of l-dopa, including nausea, vomiting, sleepiness, orthostatic hypotension, confusion, and hallucinations. Peripheral edema is common during chronic use of DAs but does not occur in patients using l-dopa as monotherapy [87]. Most side effects can be avoided by initiating treatment with small doses and titrating to therapeutic levels slowly over several weeks. Moreover, patients’ intolerance of one DA may tolerate another one. In up to 50% of patients with long-term use, DAs are associated with ICD, such as pathologic gambling, compulsive sexual behavior, or compulsive buying [89]. Dopamine receptor agonists decrease prolactin concentration [90]. Thus, a potential decreased milk production is observed in postpartum women taking these agents, which are contraindicated in women who are breastfeeding. A recent warning on Pramipexole has been issued regarding its risk of excessive somnolence, which can occur abruptly, at a dose above 1.5 mg/day [91]. Therefore, PD patients who drive may consider this adverse effect. Moreover, a DA withdrawal syndrome is described in some PD patients which abruptly stop taking a DA. A subset of patients experience physical and psychiatric disturbances, such as agitation, anxiety, diaphoresis, and drug craving, that only respond to DAs repletion. Indeed, these symptoms are refractory to other antiparkinsonian medications, including l-dopa. This phenomenon, named Dopamine Agonist Withdrawal Syndrome (DAWS), was firstly described in 2010 by Rabinak and Nirenberg [92]. In retrospective studies, frequency of DAWS is reported to range from 8% to 19% of PD patients [93].

#### 2.2.4. Ergot-Derived Dopamine Agonists

Ergot-derived DAs, as bromocriptine, cabergoline, pergolide, and lisuride, are considered a first-generation class of DAs, rarely used because of recurrent side effects. Therefore, these drugs have been largely replaced by Non-Ergot DAs. Pergolide and cabergoline have been associated with cardiac, pulmonary and peritoneal fibrosis and also valvular heart due to chronic use [94]. Those side effects are uncommon with bromocriptine, which can be used in combination with l-dopa in both early and late PD.

#### 2.2.5. Monoamine Oxidase Inhibitors

MAO-BIs represent the first step in PD treatment in order to prevent disease progression by their neuroprotective properties. In the DATATOP trial (Deprenyl and Tocopherol Antioxidative Therapy of Parkinsonism) [95] Selegiline has shown successful results in order to delay disease progression, which was calculated by the time until the patients needed potent symptomatic dopaminergic therapy. Another MAO-BI, Rasagiline, has been shown to have modest symptomatic benefits although its neuroprotective properties are still debated. In the ADAGIO trial (Attenuation of Disease Progression with Azilect Given Once-Daily) [96], it has been demonstrated that 1 mg dose of Rasagiline seems to slow disease progression. However, in a recent metanalysis, effective neuroprotective role of MAO-BI therapies was controversial and their role in a prodromal or pauci-symptomatic stage of PD is still debated.

#### 2.2.6. Anticholinergics

Anticholinergics could represent another choice in therapeutic settings, although they represent less effective anti-parkinsonian agents than DAs. However, they seem to be effective in controlling tremors and in reducing rigidity [60]. Triphexyphenidil is one of the most commonly used. The stating dose should be 2 mg three times per day, which can be gradually increased up to 15 mg or more per day. Regarding the safety profile, because of their well-known neuropsychiatric side effects due to central receptor antagonism (such as confusion, decrease short term memory, hallucinations, and psychosis) their use is generally limited to young and cognitively intact patients.

#### 2.2.7. Amantadine

Although Amantadine has been available for nearly four decades (it was originally marked as an antiviral agent), little is known about its mechanism of action. It has been thought that Amantadine has both anticholinergic and anti-glutamatergic properties and for this reason is the only antiparkinsonian drug that could improve the characteristic l-dopa induced dyskinesias often present in the latter stage of PD [97]. However, Amantadine can be useful in the first stages of PD even though its effects are more appreciable in the first months of treatment. Moreover, Amantadine use could be associated with several side effects such as visual hallucinations, ankle edema, and livedo reticularis, which can influence therapeutic compliance. The usual dose is 100 mg two times per day but dose adjustment up to 200 mg two times per day might be allowed.

## 3. Advanced Stage

During progression of PD, the beneficial effects of early stage therapies may reduce, because of dopaminergic progressive neuronal loss [15], and therefore management of MS becomes more complicated. Disabling manifestations of this advanced state include worsening of balance, falls, increasing compromise of gait, and speech disturbances [11].

Another common symptom in advanced PD is represented by dystonia. Dystonia usually follows l-dopa therapy administration and it may present with several onset patterns: during wearing off, at peak dose, or with biphasic timing. Approximately 30% of PD patients, treated with l-dopa, tend to present “off-dystonia”, especially in the morning before the first l-dopa dose. Differently from other patterns, off-dystonia is commonly painful, and the foot seems to be the main site of pain [55]. By contrast, peak-dose dystonia tends to involve the neck, face, and upper limbs [98], whereas dystonia occurring as part of diphasic dyskinesia seems to mainly involve lower limbs. Dystonia, which typically presents after years of disease, rarely occurs in an early stage, usually related to young-onset PD and to autosomal recessive genetic parkinsonism forms, such as PARK-PARKIN (PARK2) and PARK-SNCA (PARK1) mutations [99].

Nonetheless, several non-motor features may appear in the advanced stage, such as hallucinations, psychosis, dysautonomia, mood disorders, and dementia. Some NMS typically debut years before MS. However, late stages of PD are featured by adjunctive NMS, which are slighter or less common in early phases.

### 3.1. Treatments for Motor Symptoms in the Advanced Stage

In the advanced stage, treatment of MS may take advantage of a combination of more drugs. Association of tolcapone or entacapone (COMT inhibitors) with l-dopa/dopa decarboxylase inhibitor produces a prolonged duration of antiparkinsonian action, improving motor function [100]. Many cases [101,102] of hepato-toxicity have been reported with the use of tolcapone, thus practical implication of this drug has been revised as “unlikely useful”. Conversely, because of its satisfactory profile, entacapone represents the drug of choice and the triple association entacapone/l-dopa/carbidopa has become available in a single formulation (Stalevo). Some trials [103,104] have evaluated its efficacy to reduce motor fluctuation onset. Due to its tonic stimulation of dopamine receptors in the striatum, it provides more stable plasma l-dopa levels and may decrease the risk of developing motor complications (dyskinesias and “on-off” phenomenon). Recently opicapone, a new COMT inhibitors, has been approved. Two studies [105] (BIPARK 1 and 2), in which opicapone was compared with entacapone and placebo in more than 250 patients, have examined its efficacy. Indeed, it reduced by 2 h off-time and increased on-time by 1 h without raising the frequency of dyskinesia, as compared with a placebo, and this benefit was maintained for at least 1 year of therapy without increasing l-dopa dose.

Another association commonly used in advanced PD is MAO-Is with l-dopa. It is almost established that MAO-B levels are increased in PD brain as a consequence of gliosis. The blocking of MAO-B prolongs a high dopamine concentration in basal ganglia reducing the development of late complications of l-dopa treatment [106]. More recent evidence [107,108] suggests that MAO-BIs can slow down disability progression associated with PD, without substantial side effects or increased mortality. Safinamide is a new molecule characterized by a dual mechanism of action: reversible inhibition of MAO-B and modulation of glutamate release. Clinical studies have demonstrated its efficacy to control MS and motor fluctuations in a short term (about 6 months), with benefits lasting up to 2 years [109]. Furthermore, as a highly selective inhibitor, it hampers both dopamine breakdown and toxic free radical productions. In a placebo-controlled study [110] the superior benefit of Safinamide when added to a single DA has been found. It should potentiate dopaminergic activity thanks to stimulation of the D1 receptor and it could be investigated as a disease-modifying therapy in future studies because of its anti-glutamatergic properties.

Moreover, AChEI has been tested as a novel strategy to prevent falls. Previously, a study conducted on the effectiveness of Donepezil has showed a reduction in fall rate when compared with placebo, although it was characterized by a small cohort of patients and a short duration [111]. A randomized, double-blind, placebo-controlled, phase 2 trial, has demonstrated the efficacy of Rivastigmine to improve gait instability and to reduce falls in PD patients, with an acceptable safety profile [112]. However, it is necessary to undertake a larger phase 3 trial to analyze primary outcomes and costs, although the use of these agents seems to be promising in consideration of the role of cholinergic deficit in gate and cognitive dysfunction in PD patients.

Regarding dystonia, the introduction of dopamine replacement therapy (l-dopa or DAs) can determine variable effects, although some evidence has suggested a better response to DAs.

Other agents, such as Baclofen, Carbamazepine, and Benzodiazepines, may be used alone or in combination [113]. Botulinum neurotoxin, produced by Clostridium botulinum, determines local muscle weakness; hence, it is the first-line treatment for patients with blepharospasm and cervical dystonia. It also seems to be effective in laryngeal and limb dystonia [114].

### 3.2. Treatments for Non-Motor Symptoms in the Advanced Stage

#### 3.2.1. Cognitive Deficits

Cognitive deficits, by definition, should not appear within a year from MS onset (1-year rule), otherwise DLB diagnosis should be considered [115]. Over years, about 26% of PD patients will present mild cognitive impairment (MCI) features. PD-MCI is mainly associated with “subcortical” deficits (i.e., executive and attentional), but also visuospatial, praxis, language, and memory impairment can be present [116]. Dementia also appears in PD in up to 80% of cases, typically in advanced stages, and is associated with increased mortality [117].

AchEI (donepezil 5–10 mg daily, galantamine 4–8 mg twice a day, rivastigmine 1.5 mg twice a day up to 6 mg bid) can improve cognition in PD-MCI and in PDD patients [118]. Rivastigmine efficacy seems higher, especially in PDD patients, as compared to the other two drugs [60,119]. However, although AchEI does not seem to significantly increase the rate of falls in these patients, a worsening of tremor may appear. Donepezil seems to show less adverse effects, as compared to Rivastigmine, and could be employed in patients with more prominent tremor [120].

Memantine, a N methyl D aspartate (NMDA) glutamate receptor used in Alzheimer’s Disease (AD), was also investigated as a possible therapy in PD patients. Nevertheless, Memantine treated patients did not show any significant clinical improvement as compared to controls, in cognition as well as in other NMS (fatigue, apathy, mood, and quality of sleep) [121,122], so its administration is not supported by evidence. Some previous studies also assessed the effects of Rasagiline (1 mg daily) on cognitively impaired PD patients. Conflicting results have been found [123]. Nonetheless, some benefits, particularly in the executive and attentional functions, were observed in treated patients [124,125].

#### 3.2.2. Apathy

Apathy shows in 25%–29% of PD patients, who typically exhibit lack of motivation and interests, and reduced emotional reactivity [126,127]. Apathy is often confused with drug-resistant depression, due to their clinical similarity, to the untrained eye. Indeed, the two conditions require different treatments. Apathy can also be a complication following a subthalamic nucleus deep brain stimulation (STN-DBS, see later) procedure [128]. Rivastigmine (9.5 mg transdermal patch daily) was proven to be effective in reducing apathy in non-demented and non-depressed advanced PD patients [128]. DAs as Piribedil, Ropinirole, and Rotigotine, and the dopamine transporter (DaT) inhibitor methylphenidate have also demonstrated to improve apathy in post-STN-DBS apathy, in PD patients [128]. However, ICD is an NMS of PD which can also be triggered by these drugs, so caution is recommended in their administration. Antidepressant agents are not recommended to treat apathy, which could even worsen [128].

#### 3.2.3. Psychotic Disturbances

Psychotic disturbances (PsD) are quite common in PD patients. Well-formed complex visual hallucinations (VHs) represent the most common presentation of PsD, with higher incidence in advanced PD. Auditory, olfactive, and tactile hallucinations may also occur, less frequently. Paranoid delusions, Othello’s syndrome, and other kinds of delusions are other possible presentations of psychosis in PD [128,129]. Several theories have been proposed in order to explain the occurrence of these phenomena in synucleinopathies, mostly in PD and in DLB. Previous hypothesis included visual deafferentation (as in Charles-Bonnet’s syndrome), loss of cortical inhibition, adverse effects to dopamine supplementation, monoaminergic imbalance, and others [130,131]. Nowadays, a complex model has been proposed, involving different neural networks, namely Thalamocortical dysrhythmia (TCD) [132].

Pharmacological studies on VHs in synucleinopathies helped in assessing the role of the different neurotransmitters. Indeed, the importance of dopamine transmission has been significantly reduced in previous years [133,134]. Therapeutic options for PsD of course include antipsychotic agents. However, due to severe motor worsening, typical antipsychotics (e.g., haloperidol, chlorpromazine) should be avoided [135]. On the other hand, atypical antipsychotics seem to be very effective, as a possible consequence of serotonergic modulation on 5-HT2 receptors. Indeed, Clozapine has always been considered to be extremely effective in these patients [136]. However, patients receiving Clozapine must undergo weekly blood count tests. Due to this necessity, Clozapine use is now reserved to second-line treatment of PsD [137]. Quetiapine (total daily dose ranging from 25 mg up to 150–300 mg) use is nowadays very common, and this drug is often considered a first choice, due to its high tolerability [60]. Remarkably, the Food and Drug Administration (FDA) has recently warned about the increased mortality risk in elderly people receiving antipsychotics [138]. Accordingly, antipsychotics should be employed if strictly necessary.

Other novel antiserotonergic drugs have been approved to treat PsD, thus enhancing the importance of the serotonergic transmission. Pimavanserin, a selective 5-HT2A serotonin receptor inhibitor, is in fact recommended (34–40 mg daily) [139], in countries where it is available, due to its efficacy and tolerability [140,141]. Some preliminary evidence also suggest a role for Ondansetron, an anti-emetic drug, in PsD treatment [142]. However, patients receiving Quetiapine or Pimavanserin should undergo an EKG exam, due to risk of QT prolongation [60]. Other neurotransmitters have been implied in VH onset (acetylcholine, GABA, glutamate) [143]; however, current recommendations do not include therapeutic options acting on their signaling in PD patients [144,145].

#### 3.2.4. Dysphagia 

Dysphagia is a common NMS of PD, typically occurring in late stages of the disease. In fact, early and prominent dysphagia, as well as other severe bulbar dysfunction signs, represent a red flag for PD diagnosis, according to current diagnostic criteria (since it is typically associated with progressive supranuclear palsy) [3]. Lifetime prevalence of dysphagia in PD is about 80%. Only 20%–40% of patients are aware of their swallowing difficulties, which can eventually lead to ab ingestis pneumonia, but also to difficulties in medication intake, malnutrition, and dehydration [146]. Unfortunately, no specific pharmacological treatment for dysphagia is currently available. However, previous studies documented improvements in swallowing after starting LD or DAs therapy [147,148].

#### 3.2.5. Dysautonomic Symptoms

Dysautonomic symptoms (DS) occurring in advanced PD include orthostatic hypotension (OH) and urinary dysfunction (UD). Both symptoms, according to current criteria, should not be clinically relevant within the first five years of disease; otherwise, MSA diagnosis should be considered [3]. OH is a major risk factor for falls in PD patients and should be carefully evaluated. Droxidopa 100–300 mg tid, can improve lightheadedness and orthostatic dizziness as monotherapy or add-on therapy [149]. Other possible pharmacological treatments are Fludrocortisone 0.1–0.2 mg daily or (in some countries) Midodrine 10 mg tid (second-line treatment) [150]. No particular safety issues have been reported for Droxidopa, except for cardiovascular comorbidities [151,152]. However, Fludrocortisone could lead to hypokalemia and should not be administered in patients with heart or renal failure. Midodrine may cause or worsen urinary retention, instead, which is also common in advanced PD. Moreover, some degree of supine hypertension can occur with all of these drugs (less frequently with Droxidopa). Laying with a raised head could help reducing this side effect.

UD prevalence in PD patients ranges from 27% to 64%. Common presentations are overactive bladder, nocturia and, in later stages, detrusor areflexia. Micturition bradykinesia, detrusor-sphincter dyssynergia, and incomplete pelvic floor relaxation have also been observed [153]. Notably, TCAs can worsen voiding deficits in PD patients [60]. Anticholinergics can be used to reduce bladder hyperreactivity: Oxybutynin (2.5 mg bid, up to a 10–20 mg daily dose), and tolterodine (1–2 mg bid) are the most widely employed. Solifenacin (5–10 mg daily) is also considered an effective treatment for an overactive bladder and it is associated with less xerostomia [154]. Caution should be used when administering these antimuscarinic treatments, due to possible induction or worsening of cognitive impairment [155].

An alternative therapeutic option for an overactive bladder is represented by Mirabegron (50 mg daily) [156,157]. This drug acts as an agonist on β3 adrenoceptors and it has no antimuscarinic action, therefore not impairing cognition. Large phase 3 Randomized Controlled Trials have assessed the efficacy of Mirabegron monotherapy [158]. No particular recommendations for patients’ age and sex have been reported, as the molecule appears very well tolerated even for long-term therapy, also due to the lack of antimuscarinic side effects [157].

Intranasal administration of desmopressin can improve nocturia. However, the risk of severe adverse effects as cognitive impairment and hyponatremia limits the use of this drug in current clinical practice [159].

Unfortunately, no effective pharmacological treatments are currently available for the other PD-related micturition disturbances.

Non-pharmacological treatment options for NMS will be later discussed.

## 4. Complicated Stage: Pharmacological Treatment for Motor Complications

Motor fluctuations generally develop after 4–6 years of therapy and affect about 50% of PD patients. Wearing-off is the most common type, but other motor complications, such as dose failure, beginning of dose worsening, end-of-dose rebound, freezing of gait, and Levodopa-induced dyskinesia (LID), can also develop during disease progression [160,161]. Several therapeutic strategies have been adopted to reduce frequency and duration of the so-called off-periods (defined by recurrence of symptoms or lack of l-dopa effects) but none of these drugs are able to completely control motor fluctuations and their side effects may limit optimal dosage [162].

### 4.1. Motor Fluctuations

Medical treatment of motor fluctuations includes increase of l-dopa doses, increased frequency of l-dopa administration, use of modified release (MR) l-dopa and other treatments [163], involving DAs, Amantadine, MAO-Bis, and COMT inhibitors. DAs have not been shown to be effective in improving off-periods. Conversely, Apomorphine is a very effective DA that can be used both as a “rescue” therapy for off-periods, in an experimental sublingual formulation, or as a continuous infusion in advanced PD [164]. Indeed, continuous subcutaneous Apomorphine (Apo) infusion seems to be a valid strategy to contrast refractory motor complications. Apo is a D1 and D2 receptor DA, whose subcutaneous delivery improves drug bioavailability and onset of action by avoidance of gastrointestinal transit time [165]. In an RCT, to date only published in abstract form, patients receiving Apo during their waking time at up to 8 mg/h experienced a reduction in off-time of 2.47 h compared to placebo after 12 weeks. Moreover, patients had an increase in on-time without troublesome dyskinesia (TSD) [166]. Since chronic subcutaneous Apo infusion during daytime seems to improve sleep quality [167], it has been suggested that nocturnal infusion may be a well-tolerated and effective treatment to reduce insomnia and disturbing motor sleep symptoms in the advanced stage [168]. Side effects are usually transient and manageable, of which gastrointestinal disturbances are the most frequently described in literature [169]. Nausea and vomiting are frequent for acute intermittent Apo injections, used in predictable and unpredictable off-periods which requires rapid reversal, whereas they are less common in continuous infusion [170]. Tolerance to these symptoms develops rapidly [171].

COMT inhibitors have proven to be effective in wearing-off. Moreover, Opicapone seems to be more powerful, effective, and safer than other COMT inhibitors. It has also the advantage of daily single administration [172]. Opicapone consistently reduces off-time and increases on-time without rising dyskinesia frequency [173]. When most of dopaminergic projections from substantia nigra to striatum are lost, motor complications can occur due to pharmacokinetics of l-dopa itself [174]. In fact, striatal dopamine levels become strictly dependent on peripheral availability of l-dopa, whose plasma levels are fluctuating in relation to pulsatile oral administration [175]. Therefore, continuous l-dopa infusion has proven to be more similar to physiological striatal settings, reducing the occurrence and severity of dyskinesia and off-time [176]. Several novel l-dopa formulations are under investigation with innovative routes of administration (intestinal infusion, transcutaneous, or inhaled l-dopa). ND062L is a transcutaneous formulation of l-dopa/carbidopa in a patch-pump device [177]. CVT-301 is a l-dopa inhalation, used as a “rescue” therapy for sudden off: a single dose of 84 mg, administered in association with oral l-dopa/carbidopa, for early morning off symptoms seems to be well-tolerated and effective [178,179]. A randomized controlled trial (RCT) has shown that Levodopa-carbidopa intestinal gel (LCIG), administered by continuous intra-intestinal infusion (Duodopa^®^), was more effective than oral l-dopa regarding motor fluctuations. After 12 weeks on LCIG, off-time was reduced by 4 h compared to baseline and 1.91 h compared to standard oral formulation. Moreover, there was an increase in on-time without troublesome dyskinesia (TSD) [180]. Since there were no difference in UPDRS motor scores, it is unlikely that the greater reduction in off-time was attributable to disproportion in l-dopa dosing [181]. Since LCIG is delivered continuously to the proximal jejunum via percutaneous gastrojejunostomy (PEG-J), adverse effects are primarily related to surgical procedure or device, including pump malfunction, obstruction of catheter, tube displacement, and abdominal pain, usually occurring within the first 2 weeks [182]. Therefore, LCIG is efficacious but requires specialized monitoring to avoid possible complications [183]. All these therapeutic strategies are designed to increase LD bioavailability. In addition to l-dopa formulations, other drugs acting with different mechanisms are under investigation. Tozadenant, an adenosine A2A antagonist, has been conceived for motor fluctuations. Due to contrasting results, the current role of A2A antagonists in PD seem to be contradictory [184]. As previously mentioned, Safinamide seems to reduce off-periods without increasing dyskinesias, although long-term effects require further studies [185].

### 4.2. Dyskinesia

Prolonged l-dopa treatment presents a risk up to 45% for developing LID after 4–5 years. Dyskinesias appear as low-amplitude choreic movements, typically related to peak plasma concentrations of l-dopa. Their phenomenology can vary from dystonia to ballism and myoclonus, often becoming very disabling. According to their timing of onset related to l-dopa dosing, dyskinesias can be classified into peak-dose dyskinesias, off-dyskinesias, and biphasic dyskinesias. Peak-dose dyskinesias, which are the most common LID, occur generally during the highest plasma l-dopa concentration (20 min to 2 h after a dose). Off-dyskinesias, often noticed as painful foot dystonia, appear during off-states and they may be seen at first as “early-morning dystonia” [186]. Biphasic dyskinesias are less common, presenting both at the beginning and at the end of l-dopa dosing. In biphasic dyskinesias legs are mainly affected with stereotyped movements and with an evident alteration of gait. This type of LID develops mainly in male patients with early motor complications [187]. The management of LID remains challenging, requiring a delicate therapeutic balance optimizing l-dopa doses but with the risk of increased “off” time. Treatment can be based on strategies to prevent their onset, to modify dopaminergic therapy, and to provide more continuous dopaminergic stimulation using non-dopaminergic drugs [188].

Peak-dose dyskinesias are mainly treated by reducing single doses of l-dopa and adding Diphasic dyskinesias are instead the most difficult to treat. An increased dosage of l-dopa may eliminate this disturbance, but peak-dose dyskinesias usually ensue, therefore a switch to DAs is the most effective strategy. A range of emerging experimental drugs are currently under development to provide a better control of LID and other complications [189]. However, a common therapeutic strategy consists of a gradually decrease of l-dopa in each dose and in an increase l-dopa intake interval. A recent retrospective study has suggested that a less pulsatile l-dopa treatment with six doses daily was associated with a low incidence of LID in both early and advanced PD patients [190]. From pulsatile to continuous, it is also the mechanism at the basis of novel levodopa-carbidopa intestinal gel which has proven to be effective in reducing off-time and improving on-time without dyskinesia [191].

Different pathophysiological mechanisms may contribute to dyskinesia onset. As well as sensitization of dopamine receptors, also non-dopaminergic pathways, such as glutamatergic, serotoninergic and GABAergic, seem to be involved [165]. Since chronic stimulation of dopamine D1 receptors results in hyperactivation of NMDA glutamate receptors in the striatum [191], Amantadine, a nonselective NMDA antagonist, is considered useful as adjunct to l-dopa therapy for treating dyskinesias, despite its side effects which include confusion and visual hallucinations [15]. Indeed, the anti-dyskinetic effect of Amantadine has been supported by several double-blind, placebo-controlled trials [192,193]. Its long-term effects have also been investigated by Thomas and colleagues, reporting that Amantadine (300 mg) reduces dyskinesias by approximately 45%, although clinical benefits disappear by 3–8 months [194]. Thus, this evidence has allowed extended-release preparations (Amantadine ER) to be developed, recently approved by FDA in August 2017 as the first oral treatment with a proven benefit for both LID and off-time reduction [195], on the basis of two-phase III trials. The EASE LID [196] and EASE LID 3 [196] have evaluated safety and efficacy profile of Amantadine ER 274 mg at bedtime, reporting as adverse events mainly hallucinations, dry mouth, orthostatic hypotension, dizziness, and peripheral edema. In addition, FDA approved in February 2018 a combined extended and immediate-release formulation of Amantadine (Osmolex ER), indicated for drug-induced extrapyramidal reactions. Several other drugs have been tested to treat dyskinesias [197,198], even antiepileptic agents [199,200]. However, their efficacy was unsatisfactory [201], thus they are not currently recommended [202,203].

As previously discussed, the serotonin system plays such a major role in dyskinesia pathogenesis because in advanced stages of PD, serotonergic terminals tend to take up l-dopa and to convert it to dopamine. Based on this evidence, a phase I/IIa study has demonstrated the anti-dyskinetic effects of Eltoprazine (5 mg), a selective partial 5-HT1A and 5-HT1B receptor agonist [204].

### 4.3. Super-Off and Akinetic Crisis

Other motor complications of advanced PD include super-off states and akinetic crisis. These conditions share similar clinical features, including marked worsening of UPDRS-motor score, even as compared to baseline, dysphagia, and autonomic instability [205,206]. However, there are some important differences. Akinetic crisis may occur even in early PD patients and is not responsive to l-dopa rescue therapy [207,208]. Akinetic crisis is also featured by DaT-SCAN appearance of “burst striatum” (BS), which is a sign of undetectable deposition of the tracer, also present in very late stages of the disease [207,208]. Therapeutic options of super-off states include naso-gastric administration of l-dopa or DAs, endovenous Amantadine (500 mg/die) [209], Apomorphine (100–200 mg/die), and transdermal Rotigotine [210,211]. Management of complications, including hyperpyrexia, raise of CPK, and myoglobin levels and subsequent renal failure, thromboembolism, diffuse intravascular coagulation, ab ingestis pneumonia, is mandatory [212].

## 5. Non-Pharmacological Treatments for Motor and Non-Motor Symptoms

Treatment options for PD have conventionally focused on dopamine replacement to provide symptomatic relief from motor symptoms, but several adverse effects are involved. The development of new technologies and a better knowledge about the role of educational, psychological, and physical training and interventions have led to a better management of parkinsonian motor and NMS [213]. We will discuss hereby few of the most important non-pharmacological PD treatments.

### 5.1. Motor Symptoms

Education of the patients and of their caregivers is probably one of the most relevant parts of PD management and it should be performed early during disease course [214], to prevent immobilization, falls, and other complications, as well as to prevent misinformation. Indeed, a multidisciplinary approach from diagnosis is needed in order to obtain the best outcome in terms of quality of life and accumulation of disability [215]. Gastroenterologists, pneumologists, geriatricians, as well as nutritionists and physical therapists, are often needed at late stages of the disease in order to treat several complications [216]. Nonetheless, an early involvement of these professional figures may significantly improve management of the disease from the beginning [217].

Physical exercise, including aerobic walking, stretching, and physiotherapy with resistance exercises could improve gait, balance, and global physical performance in PD patients [217,218]. Specifically, it has been reported that intensive training modalities including stretching and resistance training could improve muscle strength and mobility, therefore giving a better control of some of MS [218,219], whereas aerobic activity is also helpful for cardiovascular fitness [220]. Several reports have also enlightened the improvement of non-motor disturbances from physical activity [216].

Speech therapy has been proposed as a non-pharmacological treatment for parkinsonian hypophonia and dysarthria. Although a degree of improvement is commonly observed with this therapy, a Cochrane review found the scientific evidence to be still inconclusive and postulated the need for a large, well-designed, RCT [221]. Afterwards, a more recent review pointed out the efficacy of Lee Silverman Voice Treatment (LSVT) LOUD for speech disturbances and, remarkably, also in swallowing and in facial expression [222].

Deep brain stimulation (DBS) is a well-established treatment in PD. Clinical trials have shown DBS improves motor symptoms, fluctuations, and quality of life, compared with medical therapy alone [223,224]. The target is usually in the subthalamic nucleus (STN-DBS) or in the globus pallidus internus (STN-GPi) with similar motor benefits [225], whereas thalamic DBS is also an option to treat tremors. Surgical treatment tends to be considered when motor fluctuations and dyskinesias become disabling, despite motor features continuing to respond to l-dopa. Surgical strategies used to be evaluated after 10–13 years of PD diagnosis [226]. A multicenter randomized trial has showed quality of life might be improved after STN-DBS in comparison with optimal medical therapy, if DBS was performed in an early stage of disease [227]. Despite the effectiveness of DBS, dopaminergic-resistant symptoms (i.e., axial symptoms), which poorly respond to this technique [228], can be present. Therefore, new targets, such as pedunculopontine nucleus, substantia nigra and thalamus, have been emerging as treatment of motor features [229,230].

Since the last decades, non-invasive brain stimulation (NIBS) has been explored in PD, although insufficient evidence for its use currently remain. Moreover, transcranial magnetic stimulation (rTMS) has been used to reduce motor disturbances [231]. It has been reported that high-frequency rTMS over the primary motor cortex significantly reduce subjective freezing of gait (FOG) and improve gait performance in a randomized, double-blinded, cross-over study [232]. Despite overall moderate effects of rTMS, its results are considered variable, likely related to different sites of application, stimulation frequency, and duration [189].

Transcranial Direct Current Stimulation (tDCS) is another non-invasive stimulation technique, which adopts weak constant electric currents through the scalp to modulate cortical excitability [233]. A double-blind, cross-over, randomized, sham-controlled study has shown that anodal tDCS on the primary motor cortex seems to reduce dopamine-resistant FOG in a small sample of PD patients [234]. These results have been recently confirmed [235]. However, further studies are needed to validate tDCS protocols using a larger sample of patients or an extended intervention duration [2].

Finally, physiotherapy-based exercises seem to be the most common interventions used to improve motor symptoms when compared with baseline [189].

### 5.2. Non-Motor Symptoms

As discussed, NMS of PD include several manifestations, ranging from neuropsychiatric to sleep and autonomic disturbances (see Section 2.1). Few non-pharmacological treatment options have been considered for NMS; however, evidence is often contrasting.

Cognitive behavioral therapy (CBT) is a type of talk therapy which has been shown to significantly improve several neuropsychiatric disturbances in PD patients, including anxiety, depression and ICD [60,236]. CBT is based on identifying distorted thoughts, which produce emotional discomforts, in order to replace them with better alternatives [237]. This treatment seems to ameliorate self-management skills improving patient and caregivers’ quality of lives. Therefore, an individualized multimodal approach using CBT in association with pharmacologic therapies should be considered [60,234].

Some evidence suggests cognitive stimulation therapy (CST) and cognitive rehabilitation as possible non-pharmacological interventions in PD patients with MCI [60,238]. However, further investigations are needed to identify the appropriate CST techniques for the various possible patterns and severity of cognitive impairment, also to improve compliance to the therapy [239]. 

Regarding autonomic disturbances, ensuring adequate fluid intake is mandatory for preventing orthostatic hypotension [60], as well as providing increased salt intake [240]. 

Non-motor fluctuations, sleep-related symptoms, and behavioral disturbances, seem to improve using DBS, despite no clear evidence [241]. Use of repetitive rTMS has been reported in depression, although no RCT has supported its use in PD-related depression. Moreover, anodal tDCS over the left dorsolateral prefrontal cortex seems to improve cognitive impairment in PD patients in a sham-controlled study [242]. However, a recent review has reported inconclusive outcomes for its use in PD [243]. 

## 6. Disease-Modifying Drugs: Potential Approaches 

As recent studies suggested, several pathogenic pathways are involved in PD development, including genetic mutations, apoptosis, excitotoxicity, oxidative stress, mitochondrial dysfunction, inflammation, accumulation, and toxicity of a-synuclein [244]. Therefore, several promising agents targeting different pathways have been explored in order to find neuroprotective therapies to prevent further neuronal cell loss and disease progression [245].

Currently, no disease modifying therapy has been licensed in PD yet. Previously mentioned MAO-BIs, such as Rasagiline and Selegiline, and Safinamide have been evaluated for their neuroprotective properties. Another agent examined is Coenzyme Q10 (CoQ10), which is an essential cofactor involved in mitochondrial oxidative phosphorylation, as well as a potent antioxidant. As mitochondrial dysfunction has been implicated in PD pathogenesis, it has been hypothesized that CoQ10 might have potential neuroprotective effects. Currently, there is no evidence that this agent may slow disease progression in early PD [246]. Creatine monohydrate, which is converted to phosphocreatine and can form adenosine triphosphate (ATP) transferring a phosphoryl group to adenosine diphosphate (ADP), is also implicated in mitochondrial function. Its study has shown that creatine had no statistically significant benefit compared with a placebo [247].

Regarding a-synuclein toxicity, multiple processes may be involved, including protein synthesis, mis-folding, fibril formation and aggregation, degradation, and cell-to-cell transmission. Therefore, several approaches targeting α-synuclein in PD patients have been proposed [60]. Several studies have evaluated the effects of monoclonal antibodies directed to different parts of the a-synuclein protein (N-, mid-, C-terminal, or full-length peptide). Therefore, various human clinical trials using anti-a-synuclein antibodies have been performed showing a good safety profile and tolerability, and a serum reduction of free a-synuclein levels and increased free plus antibody-bound levels has been reported [248]. Two phase 2 RCT are ongoing (RO7046015, the PASADENA trial and BIIB054, the SPARK trial) [245]. 

Nilotinib, a kinase inhibitor approved for chronic myelogenous leukemia treatment, seems to be involved in c-Abl inhibition. Its neuroprotective effect is due to a phosphorylation decrease of both parkin and a-synuclein [249]. Glucocerebrosidase gene (*GBA*) mutations are the most common genetic risk factor for PD. Moreover, it has been documented that also PD patients without GBA mutations can present lower enzymatic levels of glucocerebrosidase (GCase), likely due to a-synuclein toxicity. Therefore, another potential target might include enzyme replacement therapy. Ambroxol, a secretolytic agent approved for respiratory diseases, increases glucosylceramidase activity with effects in preclinical models [250]. 

Leucine rich-repeat kinase 2 (LRRK2) is one of the most prevalent mutations in inherited PD. Pharmacological LRRK2 inhibition has been demonstrated to bepromising in blocking α-synuclein pathology. Indeed, a recent study using LRRK2 antisense oligonucleotides (ASOs) has shown that decreased endogenous levels of LRRK2 reduces α-synuclein inclusion in order to be a potential therapeutic strategy for preventing PD [251].

Other agents, which are involved in different pathological pathways, are in advanced phases of testing in PD patients. Isradipine, approved for hypertension treatment, is a dihydropyridine calcium channel blocker with a relatively high affinity for Cav1.3 channels. Inhibition of Cav1 channels in PD seems to reduce cytosolic Ca2+ levels, mitochondrial oxidant stress, and sensitivity to toxins in neurons [252]. Unfortunately, the STEADY-PD III study has recently shown that patients who were taking isradipine did not have any difference in MS compared to the placebo [253]. Similar negative results have been recently obtained in the SURE-PD 3, a phase 3 clinical trial conducted in order to identify the role of inosine, a urate precursor, in slowing PD progression [245]. Deferiprone has an important role in oxidative stress because it is a powerful iron chelator [254]. Exenatide, approved for the treatment of diabetes mellitus type 2, is another agent that has been investigated for its potential neuroprotective effects [255]. It is a synthetic agonist for the glucagon-like peptide-1 (GLP-1) receptor, which seems to modulate different cellular processes, enhancing mitochondrial function, neurogenesis, and synaptic function and reducing inflammation [256]. Moreover, other therapies, such as caffeine [257], statins [258], nicotine [259], and physical therapy [260], might be available to modify disease progression. Indeed, although proven neuroprotective elements have not already been found in PD, different promising agents are currently being investigated.

## 7. Conclusions

PD is a disabling neurological disorder, characterized by a wide range of features with motor and non-motor manifestations. A high variability in response to therapy does not currently allow a single intervention strategy to be found. The research of biomarkers and prodromal symptoms of disease remains a primary purpose in order to obtain a long-term impact on disease progression. Moreover, additional studies focused on pathophysiological mechanisms of PD are required to select successful treatments, since effective disease modification therapies have not been identified, yet.

## Figures and Tables

**Table 1:** Main pharmacological treatments for motor and non-motor symptoms in each clinical stage of Parkinson’s Disease (PD).

**Early Stage**	
Motor Symptoms	l-dopa Non-Ergot Dopamine agonists (DA) (Pramipexole, Ropinirole, and Rotigotine) Ergot-derived Dopamine agonists (Bromocriptine, Cabergoline, Pergolide, and Lisuride) Monoamine oxidase inhibitors (Selegiline, Rasagiline) Anticholinergics (Triphexyphenidil) Amantadine
REM Sleep Behavior Disorder (RBD)	Clonazepam Melatonin
Constipation	Prebiotic fibers and probiotics Macrogol Lubiprostone
Anxiety	Benzodiazepines Buspirone SSRIs Cognitive Behavioral Therapy
Depression	SNRI (such as Venlafaxine) SSRIs
Impulse Control Disorder	Dopamine agonists discontinuation l-dopa dose adjustment
**Advanced Stage**	
Motor Symptoms	COMT inhibitors with l-dopa Monoamine oxidase inhibitors with l-dopa Safinamide
Cognitive deficits	Acetylcholinesterase inhibitors
Apathy	Rivastigmine Dopamine agonists
Psychotic disturbances	Atypical Antipsychotics (Clozapine, Quetiapine) Novel anti-serotonergic antipsychotics (Pimavanserin, eligibly Ondansetron)
Orthostatic hypotension	Droxidopa Fludrocortisone Midodrine
Urinary dysfunction	Overactive bladder: Anticholinergics (Oxybutynin, Tolterodine, and Solifenacin) and Mirabegron Nicturia: Desmopressin
**Complicated Stage**	
Motor fluctuations	Increased frequency of l-dopa administration Modified Release of l-dopa Apomorphine COMT inhibitors (Opicapone) l-dopa/Carbidopa intestinal gel
Dyskinesia	Reducing doses of l-dopa and increasing frequency Amantadine Add-on therapies (DAs, MAOB-Is, COMT-Is)
Super-off	Naso-gastric administration of l-dopa or Dopamine agonists Endovenous Amantadine Apomorphine Transdermal Rotigotine

**Table 2:** Pharmacological trials in PD: critical evaluation.

Drug	Mechanism of Action	Endpoints	Clinical Evidences	Comments
Coenzyme Q10	CoQ10 is a component of electron transport chain, which is responsible for mitochondrial adenosine triphosphate generation. It is an antioxidant that leads to decrease free radical generation. CoQ10 levels and redox status have been shown to be altered in PD patients	Change in total UPDRS score (Parts I–III) from baseline	Neither treatment groups (1200 mg/d of CoQ10 and 2400 mg/d of CoQ10) have shown any benefit compared with the placebo group	CoQ10 is safe and well tolerated, but there is no evidence of its clinical benefit
Creatine	Creatine is converted to phosphocreatine, which can transfer a phosphoryl group to adenosine diphospate (ADP) to make adenosine triphosphate (ATP)	Difference in clinical decline from baseline to 5-year follow-up in two compared groups (placebo and 10 g/dL of Creatine monohydrate)	Creatine has failed to slow the clinical progression of PD	These findings do not support the effectiveness of Creatine monohydrate in PD patients
Prasinezumab (RO7046015/PRX002)	Anti-alpha-synuclein antibody therapy	Efficacy of intravenous Prasinezumab versus placebo over 52 weeks in early in PD patients. The effectiveness is evaluated through MDS-UPDRS (Parts I–III)	Results not yet available	Ongoing (Phase 2)
BIIB054	Anti-alpha-synuclein antibody therapy	Safety and biological effects of three dosages of BIIB054 compared to placebo	Results not yet available	Ongoing (Phase 2)
Nilotinib	A c-abl inhibitor used in chronic myelocytic leukemia, which seems to decrease phosphorylation of both parkin and a-synuclein	Safety and tolerability of daily oral administration of Nilotinib	Results not yet available	Ongoing (Phase 2)
LRRK2 (leucine rich repeat kinase 2)	LRRK2 mutation is involved in inherited PD	Use of LRRK2 antisense oligonucleotides (ASOs) to decrease endogenous levels of LRRK2 and therefore to reduce α-synuclein inclusion	Administration of LRRK2 ASOs reduces LRRK2 protein levels and fibril-induced α-syn inclusions	LRRK2 ASOs is a potential therapeutic strategy for preventing PD without causing potential adverse side effects
Ambroxol	Glucocerebrosidase gene (GBA) mutations are the most common genetic risk factor for PD. Ambroxol is a secretolytic agent that seems to increase glucosylceramidase activity	Safety and tolerability of Ambroxol in PD patients	Results not yet available	Ongoing (Phase 2)
Isradipine	Isradipine is a dihydropyridine calcium channel blocker	Change in UPDRS score (Parts I–III) to evaluate long-term benefits of this drug	STEADY-PD III study has recently shown that patients treated with Isradipine did not have any difference in motor symptoms compared to placebo	
Inosine	Inosine is a urate precursor that increases plasma urate, which is the main plasma antioxidant	Rate of change in MDS-UPDRS I–III total score over 24 months	No evidence in slowing PD progression (SURE-PD 3, a phase 3 clinical trial)	
Exenatide	A glucagon-like peptide-1 (GLP-1) receptor agonist. In a preclinical model of PD, it has shown to have neuroprotective and neurorestorative effects	Improvement in off-medication MDS-UPDRS score (Part III) at 60 weeks	It has recently been discovered to have beneficial effects on motor function in a randomized, placebo-controlled trial	The difference between the two groups (Exenatide versus placebo) at 60 weeks was the same UPDRS decrease at 12 weeks, suggesting a major symptomatic effect than a disease modification
ND0612	Liquid subcutaneous formulation of l-dopa/carbidopa delivered via a pump system	- Assess the long-term safety and tolerability -Assess efficacy on daily OFF time based on home ON/OFF diaries; on the motor score and activities OFF daily living (ADL) scores of the UPDRS	Reduced daily OFF time by 2.42 ± 2.62 h and 2.13 ± 2.24 h from baseline	Ongoing (Phases 2 and 3)
*CVT-301*	l-dopa inhalation powder	Change from Pre-dose in the UPDRS Part III Score at 30 min post-dose at 12 weeks for CVT-301 high dose versus placebo	UPDRS motor score change from pre-dose to 30 min post-dose was −5.91 (SE 1.50, 95% CI −8.86 to −2.96) for the placebo group and −9.83 (1.51; −12.79 to −6.87) for the CVT-301 84 mg group (between-group difference −3.92 (−6.84 to −1.00); *p* = 0.0088)	CVT-301 (Inbrija) is approved for the intermittent treatment of OFF episodes in PD patients treated with Carbidopa/Levodopa
*NCT00660387/LCIG*	Levodopa-Carbidopa intestinal gel (LCIG), administered by continuous intra-intestinal infusion (Duodopa^®^)	- Change from baseline to week 12 in average daily normalized off-time - Change from baseline in average daily normalized on-time without troublesome dyskinesia at week 12	Reduced OFF-time after 12 weeks by 4 h compared to baseline and 1.91 h compared to standard oral formulation. Reduced OFF time by 4.04 h in LDIG group vs. 2.14 h in the standard oral formulation group (*p* = 0.0015) Increase in on-time without troublesome dyskinesia (TSD)	Approved by FDA/EU No difference in UPDRS motor scores

## References

[1] Jankovic J. (2008). Parkinson’s disease: Clinical features and diagnosis. J. Neurol. Neurosurg. Psychiatry.

[2] Connolly B.S., Lang A.E. (2014). Pharmacological treatment of Parkinson disease: A review. JAMA.

[3] Postuma R.B., Berg D., Stern M., Poewe W., Olanow C.W., Oertel W., Obeso J., Marek K., Litvan I., Lang A.E. (2015). MDS clinical diagnostic criteria for Parkinson’s disease. Mov. Disord..

[4] Gjerstad M.D., Wentzel-Larsen T., Aarsland D., Larsen J.P. (2007). Insomnia in Parkinson’s disease: Frequency and progression over time. J. Neurol. Neurosurg. Psychiatry.

[5] Walsh K., Bennett G. (2001). Parkinson’s disease and anxiety. Postgrad. Med. J..

[6] Bosboom J.L.W., Stoffers D., Wolters E.C. (2004). Cognitive dysfunction and dementia in Parkinson’s disease. J. Neural Transm..

[7] Pluck G.C., Brown R.G. (2002). Apathy in Parkinson’s disease. J. Neurol. Neurosurg. Psychiatry.

[8] Beaulieu-Boire I., Lang A.E. (2015). Behavioral effects of levodopa. Mov. Disord..

[9] Sakakibara R., Uchiyama T., Yamanishi T., Shirai K., Hattori T. (2008). Bladder and bowel dysfunction in Parkinson’s disease. J. Neural Transm..

[10] Lauzé M., Daneault J.F., Duval C. (2016). The Effects of Physical Activity in Parkinson’s Disease: A Review. J. Park. Dis..

[11] Hoehn M.M., Yahr M.D. (1967). Parkinsonism: Onset, progression and mortality. Neurology.

[12] Sveinbjornsdottir S. (2016). The clinical symptoms of Parkinson’s disease. J. Neurochem..

[13] Macleod A.D., Taylor K.S.M., Counsell C.E. (2014). Mortality in Parkinson’s disease: A systematic review and meta-analysis. Mov. Disord..

[14] Berg D., Postuma R.B., Adler C.H., Bloem B.R., Chan P., Dubois B., Gasser T., Goetz C.G., Halliday G., Joseph L. (2015). MDS research criteria for prodromal Parkinson’s disease. Mov. Disord..

[15] Braak H., Del Tredici K., Rub U., de Vos R.A., Jansen Steur E.N., Braak E. (2003). Staging of brain pathology related to sporadic Parkinson’s disease. Neurobiol. Aging.

[16] Braak H., Sastre M., Bohl J.R., de Vos R.A., Del Tredici K. (2007). Parkinson’s disease: Lesions in dorsal horn layer I, involvement of para-sympathetic and sympathetic pre-and postganglionic neurons. Acta Neuropathol..

[17] Postuma R.B., Aarsland D., Barone P., Burn D.J., Hawkes C.H., Oertel W., Ziemssen T. (2012). Identifying prodromal Parkinson’s disease: Pre-motor disorders in Parkinson’s disease. Mov. Disord..

[18] Carlsson A., Lindqvist M., Magnusson T.O.R. (1957). 3, 4-Dihydroxyphenylalanine and 5-hydroxytryptophan as reserpine antagonists. Nature.

[19] De Deurwaerdère P., Di Giovanni G., Millan M.J. (2017). Expanding the repertoire of l-DOPA’s actions: A comprehensive review of its functional neurochemistry. Prog. Neurobiol..

[20] Foley P. (2000). The l-DOPA story revisited. Further surprises to be expected?. J. Neural Transm. Suppl..

[21] Hawkes C.H., Shephard B.C., Daniel S.E. (1997). Olfactory dysfunction in Parkinson’s disease. J. Neurol. Neurosurg. Psychiatry.

[22] Jennings D., Siderowf A., Stern M., Seibyl J., Eberly S., Oakes D., Marek K., PARS Investigators (2014). Imaging prodromal Parkinson disease: The Parkinson Associated Risk Syndrome Study. Neurology.

[23] Mahlknecht P., Iranzo A., Högl B., Frauscher B., Müller C., Santamaría J., Tolosa E., Serradell M., Mitterling T., Gschliesser V. (2015). Olfactory dysfunction predicts early transition to a Lewy body disease in idiopathic RBD. Neurology.

[24] Harding A.J., Stimson E., Henderson J.M., Halliday G.M. (2002). Clinical correlates of selective pathology in the amygdala of patients with Parkinson’s disease. Brain.

[25] Doty R.L., Stern M.B., Pfeiffer C., Gollomp S.M., Hurtig H.I. (1992). Bilateral olfactory dysfunction in early stage treated and untreated idiopathic Parkinson’s disease. J. Neurol. Neurosurg. Psychiatry.

[26] Gjerde K.V., Müller B., Skeie G.O., Assmus J., Alves G., Tysnes O.B. (2018). Hyposmia in a simple smell test is associated with accelerated cognitive decline in early Parkinson’s disease. Acta Neurol. Scand..

[27] Doty R.L. (2012). Olfactory dysfunction in Parkinson disease. Nat. Rev. Neurol..

[28] De Almeida C.M.O., Pachito D.V., Sobreira-Neto M.A., Tumas V., Eckeli A.L. (2018). Pharmacological treatment for REM sleep behavior disorder in Parkinson disease and related conditions: A scoping review. J. Neurol. Sci..

[29] Mahlknecht P., Seppi K., Frauscher B., Kiechl S., Willeit J., Stockner H., Djamshidian A., Nocker M., Rastner V., Defrancesco M. (2015). Probable RBD and association with neurodegenerative disease markers: A population-based study. Mov. Disord..

[30] Iranzo A., Molinuevo J.L., Santamaría J., Serradell M., Martí M.J., Valldeoriola F., Tolosa E. (2006). Rapid-eye-movement sleep behaviour disorder as an early marker for a neurodegenerative disorder: A descriptive study. Lancet Neurol..

[31] Postuma R.B., Gagnon J.F., Vendette M., Charland K., Montplaisir J. (2008). Manifestations of Parkinson disease differ in association with REM sleep behavior disorder. Mov. Disord..

[32] Schenck C.H., Mahowald M.W. (1996). Long-term, nightly benzodiazepine treatment of injurious parasomnias and other disorders of disrupted nocturnal sleep in 170 adults. Am. J. Med..

[33] Olson E., Boeve B., Silber M. (2000). Rapid eye movement sleep behavior disorder: Demographic, clinical, and laboratory findings in 93 cases. Brain.

[34] Schenck C., Hurwitz T.D., Mahowald M.W. (1993). Symposium: Normal and abnormal REM sleep regulation: REM sleep behaviour disorder: An update on a series of 96 patients and a review of the world literature. J. Sleep Res..

[35] Aurora R.N., Zak R.S., Maganti R.K., Auerbach S.H., Casey K.R., Chowdhuri S., Karippot A., Ramar K., Kristo D.A., Morgenthaler T.I. (2010). Best practice guide for the treatment of REM sleep behavior disorder (RBD). J. Clin. Sleep Med..

[36] Brzezinski A. (1997). Melatonin in humans. N. Engl. J. Med..

[37] Mcarter S.J., Boswell C.L., St Louis E., Dueffert L.G., Slocumb N., Boeve B.F., Silber M.H., Olson E.J., Tippmann-Peikert M. (2013). Treatment outcomes in REM sleep behavior disorder. Sleep Med..

[38] Takeuchi N., Uchimura N., Hashizume Y., Mukai M., Etoh Y., Yamamoto K., Kotorii T., Ohshima H., Ohshima M., Maeda H. (2001). Melatonin therapy for REM sleep behavior disorder. Psychiatry Clin. Neurosci..

[39] Boeve B.F., Silber M.H., Ferman T.J. (2003). Melatonin for treatment of REM sleep behavior disorder in neurologic disorders: Results in 14 patients. Sleep Med..

[40] Kunz D., Mahlberg R. (2010). A two-part, double-blind, placebo-controlled trial of exogenous melatonin in REM sleep behaviour disorder. J. Sleep Res..

[41] Bonakis A., Economou N.T., Papageorgiou S.G., Vagiakis E., Nanas S., Paparrigopoulos T. (2012). Agomelatine may improve REM sleep behavior disorder symptoms. J. Clin. Psychopharmacol..

[42] Kashihara K., Nomura T., Maeda T., Tsuboi Y., Mishima T., Takigawa H., Nakashima K. (2016). Beneficial Effects of Ramelteon on Rapid Eye Movement Sleep Behavior Disorder Associated with Parkinson’s Disease—Results of a Multicenter Open Trial. Intern. Med..

[43] Garcia-Borreguero D., Caminero A.B., de la Llave Y., Larrosa O., Barrio S., Granizo J.J., Pareja J.A. (2002). Decreased phasic EMG activity during rapid eye movement sleep in treatment-naive Parkinson’s disease: Effects of treatment with levodopa and progression of illness. Mov. Disord..

[44] Ozekmekci S., Apaydin H., Kilic E. (2005). Clinical features of 35 patients with Parkinson’s disease displaying REM behavior disorder. Clin. Neurol. Neurosurg..

[45] Fantini M.L., Gagno J.-F., Filipini D., Montplaisir J. (2003). The effects of pramipexole in REM sleep behavior disorder. Neurology.

[46] Sasai T., Matsuura M., Inoue Y. (2013). Factors associated with the effect of pramipexole on symptoms of idiopathic REM sleep behavior disorder. Park. Relat. Disord..

[47] Kumru H., Iranzo A., Carrasco E., Valldeoriola F., Marti M.J., Santamaria J., Tolosa E. (2008). Lack of effects of pramipexole on REM sleep behavior disorder in Parkinson disease. Sleep.

[48] Rye D.B. (1997). Contributions of the pedunculopontine region to normal and altered REM sleep. Sleep.

[49] Hendricks J.C., Morrison A.R., Mann G.L. (1982). Different behaviors during paradoxical sleep without atonia depend on pontine lesion site. Brain Res..

[50] Greene R.W., Gerber U., Carley R.W. (1989). Cholinergic activation of medial pontine reticular formation neurons in vitro. Brain Res..

[51] Boeve B.F., Silber M.H., Saper C.B., Ferman T.J., Dickson D.W., Parisi J.E., Benarroch E.E., Ahlskog J.E., Smith G.E., Caselli R.C. (2007). Pathophysiology of REM sleep behaviour disorder and relevance to neurodegenerative disease. Brain..

[52] Larsson V., Aarsland D., Ballard C., Minthon L., Londos E. (2010). The effect of memantine on sleep behaviour in dementia with Lewy bodies and Parkinson’s disease dementia. Int. J. Geriatr. Psychiatry.

[53] Shneerson J.M. (2009). Successful treatment of REM sleep behavior disorder with sodium oxybate. Clin. Neuropharmacol..

[54] Moghadam K.K., Pizza F., Primavera A., Ferri R., Plazzi G. (2017). Sodium oxybate for idiopathic REM sleep behavior disorder: A report on two patients. Sleep Med..

[55] Chagas M.H.N., Eckeli A.L., Zuardi A.W., Pena-Pereira M.A., Sobreira-Neto M.A., Sobreira E.T., Camilo M.R., Bergamaschi M.M., Schenck C.H., Hallak J.E. (2014). Cannabidiol can improve complex sleep-related behaviours associated with rapid eye movement sleep behaviour disorder in Parkinson’s disease patients: A case series. J. Clin. Pharm..

[56] Edwards L.L., Pfeiffer R.F., Quigley E.M., Hofman R., Balluff M. (1991). Gastrointestinal symptoms in Parkinson’s disease. Mov. Disord..

[57] Savica R., Carlin J.M., Grossardt B.R., Bower J.H., Ahlskog J.E., Maraganore D.M., Bharucha A.E., Rocca W.A. (2009). Medical records documentation of constipation preceding Parkinson disease: A case-control study. Neurology.

[58] Pedrosa Carrasco A.J., Timmermann L., Pedrosa D.J. (2018). Management of constipation in patients with Parkinson’s disease. NPJ Park. Dis..

[59] Seppi K., Ray Chaudhuri K., Coelho M., Fox S.H., Katzenschlager R., Perez Lloret S., Weintraub D., Sampaio C., the collaborators of the Parkinson’s Disease Update on Non-Motor Symptoms Study Group on behalf of the Movement Disorders Society Evidence-Based Medicine Committee (2019). Update on treatments for nonmotor symptoms of Parkinson’s disease-an evidence-based medicine review. Mov. Disord..

[60] Muller B., Assmus J., Larsen J.P., Haugarvoll K., Skeie G.O., Tysnes O.B., ParkWest study group (2013). Autonomic symptoms and dopaminergic treatment in de novo Parkinson’s disease. Acta Neurol. Scand..

[61] Pagano G., Tan E.E., Haider J.M., Bautista A., Tagliati M. (2015). Constipation is reduced by beta-blockers and increased by dopaminergic medications in Parkinson’s disease. Park. Relat. Disord..

[62] Tateno F., Sakakibara R., Yokoi Y., Kishi M., Ogawa E., Uchiyama T., Yamamoto T., Yamanishi T., Takahashi O. (2011). Levodopa ameliorated anorectal constipation in de novo Parkinson’s disease: The Ql-GAT study. Park. Relat. Disord..

[63] Barone P. (2010). Neurotransmission in Parkinson’s disease: Beyond dopamine. Eur. J. Neurol..

[64] Santamaria J., Tolosa E., Valles A. (1986). Parkinson’s disease with depression: A possible subgroup of idiopathic parkinsonism. Neurology.

[65] Lieberman A. (2006). Depression in Parkinson’s Disease—Review. Acta Neurol. Scand..

[66] Henderson R., Kurlan R., Kerson J.M., Como P. (1992). Preliminary examination of the comorbidity of anxiety and depression in Parkinson’s disease. J. Neuropsychiatry Clin. Neurosci..

[67] Cipriani A., Furukawa T.A., Salanti G., Chaimani A., Atkinson L.Z., Ogawa Y., Leucht S., Ruhe H.G., Turner E.H., Higgins J.P.T. (2018). Comparative efficacy and acceptability of 21 antidepressant drugs for the acute treatment of adults with major depressive disorder: A systematic review and network meta-analysis. Lancet.

[68] Seppi K., Weintraub D., Coelho M., Perez-Lloret S., Fox S.H., Katzenschlager R., Hametner E.M., Poewe W., Rascol O., Goetz C.G. (2011). The Movement Disorder Society evidence-based medicine review update: Treatments for the non-motor symptoms of Parkinson’s disease. Mov. Disord..

[69] Poewe W. (2007). Depression in Parkinson’s Disease. J. Neurol..

[70] Rana A.Q., Ahmed U.S., Chaudry Z.M., Vasan S. (2015). Parkinson’s disease: A review of non-motor symptoms. Expert Rev. Neurother..

[71] Martinez-Castrillo J.C. (2019). Impulse control disorders in Parkinson’s disease: A hard-turning point. J. Neurol. Neurosurg. Psychiatry.

[72] Molde H., Moussavi Y., Kopperud S.T., Erga A.H., Hansen A.L., Pallesen S. (2018). Impulse-Control Disorders in Parkinson’s Disease: A Meta-Analysis and Review of Case–Control Studies. Front. Neurol..

[73] Pondal M., Marras C., Miyasaki J., Moro E., Armstrong M.J., Strafella A.P., Shah B.B., Fox S., Prashanth L.K., Phielipp N. (2012). Clinical features of dopamine agonist withdrawal syndrome in a movement disorders clinic. J. Neurol. Neurosurg. Psychiatry.

[74] Isaacson S.I., Hauser R.A. (2009). Improving Symptom Control in Early Parkinson’s Disease. Ther. Adv. Neurol. Disord..

[75] Alexander T., Sortwell C.E., Sladek C.D., Roth R.H., Steece-Collier K. (1997). Comparison of neurotoxicity following repeated administration of l-dopa, D-dopa and dopamine to embryonic mesencephalic dopamine neurons in cultures derived from Fisher 344 and Sprague-Dawley donors. Cell Transplant..

[76] Fahn S. (1999). Parkinson disease, the effect of levodopa, and the ELLDOPA trial. Earlier vs Later l-DOPA. Arch. Neurol..

[77] Glover A., Ghilardi M.F., Bodis-Wollner I., Onofrj M. (1988). Alterations in event-related potentials (ERPs) of MPTP-treated monkeys. Electroencephalogr. Clin. Neurophysiol..

[78] Ghilardi M.F., Chung E., Bodis-Wollner I., Dvorzniak M., Glover A., Onofrj M. (1988). Systemic 1-methyl,4-phenyl,1-2-3-6-tetrahydropyridine (MPTP) administration decreases retinal dopamine content in primates. Life Sci..

[79] Pakkenberg H., Birket-Smith E., Dupont E., Hansen E., Mikkelsen B., Presthus J., Rautakorpi I., Riman E., Rinne U.K. (1976). Parkinson’s disease treated with Sinemet or Madopar. A controlled multicenter trial. Acta Neurol. Scand..

[80] Filograna R., Beltramini M., Bubacco L., Bisaglia M. (2016). Anti-Oxidants in Parkinson’s Disease Therapy: A Critical Point of View. Curr. Neuropharmacol..

[81] Park H.J., Kang J.K., Lee M.K. (2019). 1-O-Hexyl-2,3,5-Trimethylhydroquinone Ameliorates l-DOPA-Induced Cytotoxicity in PC12 Cells. Molecules.

[82] Yu X.X., Fernandez H.H. (2017). Dopamine agonist withdrawal syndrome: A comprehensive review. J. Neurol. Sci..

[83] Walkinshaw G., Waters C.M. (1995). Induction of apoptosis in catecholaminergic PC12 cells by l-DOPA. Implications for the treatment of Parkinson’s disease. J. Clin. Investig..

[84] Borovac J.A. (2016). Side effects of a dopamine agonist therapy for Parkinson’s disease: A mini-review of clinical pharmacology. Yale J. Biol. Med..

[85] Stowe R.L., Ives N.J., Clarke C., van Hilten J., Ferreira J., Hawker R.J., Shah L., Wheatley K., Gray R. (2008). Dopamine agonist therapy in early Parkinson’s disease. Cochrane Database Syst. Rev..

[86] Hubble J.P., Koller W.C., Cutler N.R., Sramek J.J., Friedman J., Goetz C., Ranhosky A., Korts D., Elvin A. (1995). Pramipexole in patients with early Parkinson’s disease. Clin. Neuropharmacol..

[87] Pezzoli G., Martignoni E., Pacchetti C., Angeleri V.A., Lamberti P., Muratorio A., Bonuccelli U., De Mari M., Foschi N., Cossutta E. (1994). Pergolide compared with bromocriptine in Parkinson’s disease: A multicenter, crossover, controlled study. Mov. Disord..

[88] (2019). Mirapex (pramipexole dihydrochloride) [product monograph].

[89] Kassubek J., Abler B., Pinkhardt E.H. (2011). Neural reward processing under dopamine agonists: Imaging. J. Neurol. Sci..

[90] Schilling J.C., Adamus W.S., Palluk R. (1992). Neuroendocrine and side effect profile of pramipexole, a new dopamine receptor agonist, in humans. Clin. Pharm..

[91] Tholfsen L.K., Larsen J.P., Schulz J., Tysnes O.B., Gjerstad M.D. (2015). Development of excessive daytime sleepiness in early Parkinson disease. Neurology.

[92] Rabinak C.A., Nirenberg M.J. (2010). Dopamine agonist withdrawal syndrome in Parkinson disease. Arch. Neurol..

[93] Patel S., Garcia X., Mohammad M.E., Yu X.X., Vlastaris K., O’Donnell K., Sutton K., Fernandez H.H. (2016). Dopamine agonist withdrawal syndrome (DAWS) in a tertiary Parkinson’s disease center. Mov. Disord..

[94] Zanettini R., Antonini A., Gatto G., Gentile R., Tesei S., Pezzoli G. (2007). Valvular heart disease and the use of dopamine agonists for Parkinson’s disease. N. Engl. J. Med..

[95] Parkinson Study Group (1989). DATATOP: A multicenter controlled clinical trial in early Parkinson’s disease. Arch. Neurol..

[96] Olanow C.W., Rascol O., Hauser R., Feigin P.D., Jankovic J., Lang A., Langston W., Melamed E., Poewe W., Stocchi F. (2009). A double-blind, delayed-start trial of rasagiline in Parkinson’s disease. N. Engl. J. Med..

[97] Crosby N., Deane K.H., Clarke C.E. (2003). Amantadine in Parkinson’s disease. Cochrane Database Syst. Rev..

[98] Shetty A.S., Bhatia K.P., Lang A.E. (2019). Dystonia and Parkinson’s disease: What is the relationship?. Neurobiol. Dis..

[99] Niemann N., Jankovic J. (2019). Juvenile parkinsonism: Differential diagnosis, genetics, and treatment. Park. Relat. Disord..

[100] Davie C.A. (2008). A Review of Parkinson’s disease. Br. Med. Bull..

[101] Borges N. (2005). Tolcapone in Parkinson’s disease: Liver toxicity and clinical efficacy. Expert Opin. Drug Saf..

[102] Warre C. (2000). Tolcapone and Hepatotoxic effects. Arch. Neurol..

[103] Koller W.C., Hutton J.T., Tolosa E., Capilldeo R. (1999). Immediate-release and controlled-release carbidopa/levodopa in PD: A 5-year randomized multicenter study. Carbidopa/Levodopa Study Group. Neurology.

[104] Nissinen H., Kuoppama M., Leinonen M., Schapira A.H. (2009). Early versus delayed initiation of entacapone in levodopa-treated patients with Parkinson_s disease: A long-term, retrospective analysis. Eur. J. Neurol..

[105] Less A.J., Ferrera J., Poewe W., Rocha J.F., McCrory M., Soares-da-Silva P., BIPARK-2 Study Investigators (2017). Opicapone as adjunct to levodopa therapy in patients with Parkinson’s disease and motor fluctuation: A randomized clinical trial. JAMA Neurol..

[106] Rascol O., Oertel W., Poewe W., Stocchi F., Tolosa E., LARGO study group (2005). Rasagiline as an adjunct to levodopa in patients with Parkinson’s disease and motor fluctuations (LARGO, Lasting effect in Adjunct therapy with Rasagiline Given Once daily, study): A randomised, double-blind, parallel-group trial. Lancet.

[107] Parkinson Study Group (1996). Effect of lazabemide on the progression of disability in early Parkinson’s disease. Ann. Neurol..

[108] Ives N.J., Stowe R.L., Marro J., Counsell C., Macleod A., Clarke C.E., Gray R., Wheatley K. (2004). Monoamine oxidase type B inhibitors in early Parkinson’s disease: Meta-analysis of 17 randomised trials involving 3525 patients. BMJ.

[109] Borgohain R., Szasz J., Stanzione P., Meshram C., Bhatt M., Chirilineau D., Stocchi F., Lucini V., Giuliani R., Forrest E. (2014). Randomized trial of safinamide add-on to levodopa in Parkinson’s disease with motor fluctuations. Mov. Disord..

[110] Stocchi F., Arnold G., Onofrj M., Kwiecinski H., Szczudlik A., Thomas A., Bonuccelli U., Van Dijk A., Cattaneo C., Sala P. (2004). Improvement of motor function in early Parkinson’s disease by safinamide. Neurology.

[111] Chung K.A., Lobb B.M., Nutt J.G., Horak F.B. (2010). Effects of a central cholinesterase inhibitor on reducing falls in Parkinson disease. Neurology.

[112] Henderson E.J., Lord S.R., Brodie M.A., Gaunt D.M., Lawrence A.D., Close J.C., Whone A.L., Ben-Shlomo Y. (2016). Rivastigmine for gait stability in patients with Parkinson’s disease (ReSPonD): A randomised, double-blind, placebo-controlled, phase 2 trial. Lancet.

[113] Jankovic J. (2006). Treatment of dystonia. Lancet Neurol..

[114] Albanese A., Asmus F., Bhatia K.P., Elia A.E., Elibol B., Filippini G., Gasser T., Krauss J.K., Nardocci N., Newton A. (2011). EFNS guidelines on diagnosis and treatment of primary dystonias. Eur. J. Neurol..

[115] McKeith I.G., Boeve B.F., Dickson D.W., Halliday G., Taylor J.P., Weintraub D., Aarsland D., Galvin J., Attems J., Ballard C.G. (2017). Diagnosis and management of dementia with Lewy bodies. Neurology.

[116] Litvan I., Aarsland D., Adler C.H., Goldman J.G., Kulisevsky J., Mollenhauer B., Rodriguez-Oroz M.C., Tröster A.I., Weintraub D. (2011). MDS task force on mild cognitive impairment in Parkinson’s disease: Critical review of PD-MCI. Mov. Disord..

[117] Levy G., Tang M.X., Louis E.D., Côté L.J., Alfaro B., Mejia H., Stern Y., Marder K. (2002). The association of incident dementia with mortality in, P.D. Neurology.

[118] Rolinski M., Fox C., Maidment I., McShane R. (2012). Cholinesterase inhibitors for dementia with Lewy bodies, Parkinson’s disease dementia and cognitive impairment in Parkinson’s disease. Cochrane Database Syst. Rev..

[119] Mamikonyan E., Xie S.X., Melvin E., Weintraub D. (2015). Rivastigmine for mild cognitive impairment in Parkinson disease: A placebo-controlled study. Mov. Disord..

[120] Pagano G., Rengo G., Pasqualetti G., Femminella G.D., Monzani F., Ferrara N., Tagliati M. (2015). Cholinesterase inhibitors for Parkinson’s disease: A systematic review and meta-analysis. J. Neurol. Neurosurg. Psychiatry.

[121] Ondo W.G., Shinawi L., Davidson A., Lai D. (2011). Memantine for non-motor features of Parkinson’s disease: A double-blind placebo controlled exploratory pilot trial. Park. Relat. Disord..

[122] Emre M., Tsolaki M., Bonuccelli U., Destée A., Tolosa E., Kutzelnigg A., Ceballos-Baumann A., Zdravkovic S., Bladström A., Jones R. (2010). Memantine for patients with Parkinson’s disease dementia or dementia with Lewy bodies: A randomised, double-blind, placebo-controlled trial. Lancet Neurol..

[123] Weintraub D., Hauser R.A., Elm J.J., Pagan F., Davis M.D., Choudhry A., MODERATO Investigators (2016). Rasagiline for mild cognitive impairment in Parkinson’s disease: A placebo-controlled trial. Mov. Disord..

[124] Hanagasi H.A., Gurvit H., Unsalan P., Horozoglu H., Tuncer N., Feyzioglu A., Gunal D.I., Yener G.G., Cakmur R., Sahin H.A. (2011). The effects of rasagiline on cognitive deficits in Parkinson’s disease patients without dementia: A randomized, double-blind, placebo-controlled, multicenter study. Mov. Disord..

[125] Frakey L.L., Friedman J.H. (2017). Cognitive Effects of Rasagiline in Mild-to-Moderate Stage Parkinson’s Disease Without Dementia. J. Neuropsychiatry Clin. Neurosci..

[126] Pagonabarraga J., Kulisevsky J. (2017). Apathy in Parkinson’s Disease. Nonmotor Park. Hidden Face Many Hidden Faces.

[127] Devos D., Moreau C., Maltete D., Lefaucheur R., Kreisler A., Eusebio A., Defer G., Ouk T., Azulay J.P., Krystkowiak P. (2013). Rivastigmine in apathetic but dementia and depression-free patients with Parkinson’s disease: A double-blind, placebo-controlled, randomised clinical trial. J. Neurol. Neurosurg. Psychiatry.

[128] Ravina B., Marder K., Fernandez H.H., Friedman J.H., McDonald W., Murphy D., Aarsland D., Babcock D., Cummings J., Endicott J. (2007). Diagnostic criteria for psychosis in Parkinson’s disease: Report of an NINDS, NIMH work group. Mov. Disord..

[129] Ojo O.O., Fernandez H.H. (2016). Current understanding of psychosis in Parkinson’s disease. Curr. Psychiatry Rep..

[130] Onofrj M., Carrozzino D., D’Amico A., Di Giacomo R., Delli Pizzi S., Thomas A., Onofrj V., Taylor J.P., Bonanni L. (2017). Psychosis in parkinsonism: An unorthodox approach. Neuropsychiatr. Dis. Treat..

[131] Perry E.K., Marshall E., Kerwin J., Smith C.J., Jabeen S., Cheng A.V., Perry R. (1990). Evidence of a monoaminergic-cholinergic imbalance related to visual hallucinations in Lewy body dementia. J. Neurochem..

[132] Llinas R.R., Ribary U., Jeanmonod D., Kronberg E., Mitra P.P. (1999). Thalamocortical dysrhythmia: A neurological and neuropsychiatric syndrome characterized by magnetoencephalography. Proc. Natl. Acad. Sci. USA.

[133] García Ruiz P.J., Sesar Ignacio A., Ares Pensado B., Castro García A., Alonso Frech F., Alvarez López M., Arbelo González J., Baiges Octavio J., Burguera Hernández J.A., Calopa Garriga M. (2008). Efficacy of long-term continuous subcutaneous apomorphine infusion in advanced Parkinson’s disease with motor fluctuations. Mov. Disord..

[134] Borgemeester R.W., Drent M., van Laar T. (2016). Motor and non-motor outcomes of continuous apomorphine infusion in 125 Parkinson’s disease patients. Park. Relat. Disord..

[135] Aarsland D., Perry R., Larsen J.P., McKeith I.G., O’Brien J.T., Perry E.K., Burn D., Ballard C.G. (2005). Neuroleptic sensitivity in Parkinson’s disease and parkinsonian dementias. J. Clin. Psychiatry.

[136] Eng M.L., Welty T.E. (2010). Management of hallucinations and psychosis in Parkinson’s disease. Am. J. Geriatr. Pharmacother..

[137] Tuunainen A., Wahlbeck K., Gilbody S. (2002). Newer atypical antipsychotic medication in comparison to clozapine: A systematic review of randomized trials. Schizophr. Res..

[138] Maust D.T., Kim H.M., Seyfried L.S., Chiang C., Kavanagh J., Schneider L.S., Kales H.C. (2015). Antipsychotics, other psychotropics, and the risk of death in patients with dementia: Number needed to harm. JAMA Psychiatry.

[139] Meltzer H.Y., Mills R., Revell S., Williams H., Johnson A., Bahr D., Friedman J.H. (2009). Pimavanserin, a Serotonin2A Receptor Inverse Agonist, for the Treatment of Parkinson’s Disease Psychosis. Neuropsychopharmacology.

[140] Cummings J., Isaacson S., Mills R., Williams H., Chi-Burris K., Corbett A., Dhall R., Ballard C. (2014). Pimavanserin for patients with Parkinson’s disease psychosis: A randomised, placebo-controlled phase 3 trial. Lancet.

[141] Combs B.L., Cox A.G. (2017). Update on the treatment of Parkinson’s disease psychosis: Role of pimavanserin. Neuropsychiatr. Dis. Treat..

[142] Zoldan J., Friedberg G., Livneh M., Melamed E. (1995). Psychosis in advanced Parkinson’s disease: Treatment with ondansetron, a 5-HT3 receptor antagonist. Neurology.

[143] Khundakar A.A., Hanson P.S., Erskine D., Lax N.Z., Roscamp J., Karyka E., Tsefou E., Singh P., Cockell S.J., Gribben A. (2016). Analysis of primary visual cortex in dementia with Lewy bodies indicates GABAergic involvement associated with recurrent complex visual hallucinations. Acta Neuropathol. Commun..

[144] Minett T., Thomas A., Wilkinson L.M., Daniel S.L., Sanders J., Richardson J., Littlewood E., Myint P., Newby J., McKeith I.G. (2003). What happens when donepezil is suddenly withdrawn? An open label trial in dementia with Lewy bodies and Parkinson’s disease with dementia. Int. J. Geriatr. Psychiatry.

[145] Riederer P., Lange K.W., Kornhuber J., Danielczyk W. (1991). Pharmacotoxic psychosis after memantine in Parkinson’s disease. Lancet.

[146] Suttrup I., Warnecke T. (2015). Dysphagia in Parkinson’s Disease. Dysphagia.

[147] Lim A., Leow L., Huckabee M.L., Frampton C., Anderson T. (2008). A pilot study of respiration and swallowing integration in Parkinson’s disease: “on” and “off” levodopa. Dysphagia.

[148] Hirano M., Isono C., Sakamoto H., Ueno S., Kusunoki S., Nakamura Y. (2015). Rotigotine Transdermal Patch Improves Swallowing in Dysphagic Patients with Parkinson’s Disease. Dysphagia.

[149] Kremens D., Lew M., Claassen D., Goodman B.P. (2017). Adding droxidopa to fludrocortisone or midodrine in a patient with neurogenic orthostatic hypotension and Parkinson disease. Clin. Auton. Res..

[150] Wu C.K., Hohler A.D. (2014). Management of orthostatic hypotension in patients with Parkinson’s disease. Pract. Neurol..

[151] Hauser R.A., Biaggioni I., Hewitt L.A., Vernino S. (2018). Integrated Analysis of Droxidopa for the Treatment of Neurogenic Orthostatic Hypotension in Patients with Parkinson Disease. Mov. Disord. Clin. Pract..

[152] Biaggioni I., Freeman R., Mathias C.J., Low P., Hewitt L.A., Kaufmann H., Droxidopa 302 Investigators (2014). Randomized Withdrawal Study of Patients with Symptomatic Neurogenic Orthostatic Hypotension Responsive to Droxidopa—Novelty and Significance. Hypertension.

[153] Yeo L., Singh R., Gundeti M., Barua J.M., Masood J. (2011). Urinary tract dysfunction in Parkinson’s disease: A review. Int. Urol. Nephrol..

[154] Madhuvrata P., Cody J.D., Ellis G., Herbison G.P., Hay-Smith E.J. (2012). Which anticholinergic drug for overactive bladder symptoms in adults. Cochrane Database Syst. Rev..

[155] Winge K., Fowler C.J. (2006). Bladder dysfunction in Parkinsonism: Mechanisms, prevalence, symptoms, and management. Mov. Disord..

[156] Chapple C.R. (2017). Mirabegron for the treatment of overactive bladder: A review of efficacy, safety and tolerability with a focus on male, elderly and antimuscarinic poor-responder populations, and patients with OAB in Asia. J. Expert Rev. Clin. Pharmacol..

[157] Deeks E.D. (2018). Mirabegron: A Review in Overactive Bladder Syndrome. Drugs.

[158] Rossanese M., Novara G., Challacombe B., Iannetti A., Dasgupta P., Ficarra V. (2015). Critical analysis of phase II and III randomised control trials (RCTs) evaluating efficacy and tolerability of a β_3_-adrenoceptor agonist (Mirabegron) for overactive bladder (OAB). BJU Int..

[159] Palma J.A., Kaufmann H. (2018). Treatment of Autonomic Dysfunction in Parkinson Disease and Other Synucleinopathies. Mov. Disord..

[160] Dewey R.B. (2004). Management of motor complications in Parkinson’s disease. Neurology.

[161] Freitas M.E., Hess C.W., Fox S.H. (2017). Motor Complications of Dopaminergic Medications in Parkinson’s Disease. Semin. Neurol..

[162] Morgan J.C., Fox S.H. (2016). Treating the Motor Symptoms of Parkinson Disease. Continuum.

[163] Cabreira V., Soares-da-Silva P., Massano J. (2019). Contemporary Options for the Management of Motor Complications in Parkinson’s Disease: Updated Clinical Review. Drugs.

[164] Trosch R.M., Silver D., Bottini P.B. (2008). Intermittent subcutaneous apomorphine therapy for ‘off’ episodes in Parkinson’s disease: A 6-month open-label study. CNS Drugs.

[165] Papuć E., Trzciniecka O., Rejdak K. (2019). Continuous subcutaneous apomorphine monotherapy in Parkinson’s disease. Ann. Agric. Environ. Med..

[166] Katzenschlager R., Poewe W., Rascol O., Trenkwalder C., Deuschl G., Chaudhuri R., Henriksen T., van Laar T., Spivey K., Vel S. (2017). Double-blind, randomized, placebo-controlled, Phase III study (TOLEDO) to evaluate the efficacy of apomorphine subcutaneous infusion in reducing OFF time in Parkinson’s disease patients with motor fluctuations not well controlled on optimized medical treatment. Neurology.

[167] Martinez-Martin P., Reddy P., Antonini A., Henriksen T., Katzenschlager R., Odin P., Todorova A., Naidu Y., Tluk S., Chandiramani C. (2011). Chronic subcutaneous infusion therapy with apomorphine in advanced Parkinson’s disease compared to conventional therapy: A real life study of non motor effect. J. Park. Dis..

[168] Fernández-Pajarín G., Sesar Á., Ares B., Castro A. (2016). Evaluating the efficacy of nocturnal continuous subcutaneous apomorphine infusion in sleep disorders in advanced parkinson’s disease: The APO-NIGHT study. J. Park. Dis..

[169] Gancher S.T., Woodward W.R., Boucher B., Nutt J.G. (1989). Peripheral pharmacokinetics of apomorphine in humans. Ann. Neurol..

[170] Chen J.J., Obering C. (2005). A review of intermittent subcutaneous apomorphine injections for the rescue management of motor fluctuations associated with advanced Parkinson’s disease. Clin. Ther..

[171] Hughes A.J., Bishop S., Kleedorfer B., Turjanski N., Fernandez W., Lees A.J., Stern G.M. (1993). Subcutaneous apomorphine in Parkinson’s disease: Response to chronic administration for up to five years. Mov. Disord..

[172] Rodrigues F.B., Ferreira J.J. (2017). Opicapone for the treatment of Parkinson’s disease. Expert Opin. Pharm..

[173] Ferreira J.J., Lees A., Rocha J.F., Poewe W., Rascol O., Soares-da-Silva P. (2019). Long-term efficacy of opicapone in fluctuating Parkinson’s disease patients: A pooled-analysis of data from two Phase 3 clinical trials and their open-label extensions. Eur. J. Neurol..

[174] Obeso J.A., Rodriguez-Oroz M., Marin C., Alonso F., Zamarbide I., Lanciego J.L., Rodriguez-Diaz M. (2004). The origin of motor fluctuations in Parkinson’s disease: Importance of dopaminergic innervation and basal ganglia circuits. Neurology.

[175] LeWitt P.A. (2015). Levodopa therapy for Parkinson’s disease: Pharmacokinetics and pharmacodynamics. Mov. Disord..

[176] Antonini A., Odin P. (2009). Pros and cons of apomorphine and l-dopa continuous infusion in advanced Parkinson’s disease. Park. Relat. Disord..

[177] Freitas M.E., Ruiz-Lopez M., Fox S.H. (2016). Novel Levodopa Formulations for Parkinson’s Disease. CNS Drugs.

[178] Hauser R.A., Isaacson S.H., Ellenbogen A., Safirstein B.E., Truong D.D., Komjathy S.F., Kegler-Ebo D.M., Zhao P., Oh C. (2019). Orally inhaled levodopa (CVT-301) for early morning OFF periods in Parkinson’s disease. Park. Relat Disord..

[179] LeWitt P.A., Hauser R.A., Pahwa R., Isaacson S.H., Fernandez H.H., Lew M., Saint-Hilaire M., Pourcher E., Lopez-Manzanares L., Waters C. (2019). Safety and efficacy of CVT-301 (levodopa inhalation powder) on motor function during off periods in patients with Parkinson’s disease: A randomised, double-blind, placebo-controlled phase 3 trial. Lancet Neurol..

[180] Timpka J., Mundt-Petersen U., Odin P. (2016). Continuous dopaminergic stimulation therapy for Parkinson’s disease–recent advances. Curr. Opin. Neurol..

[181] Antonini A., Fung V.S., Boyd J.T., Slevin J.T., Hall C., Chatamra K., Eaton S., Benesh J.A. (2016). Effect of levodopa-carbidopa intestinal gel on dyskinesia in advanced Parkinson’s disease patients. Mov. Disord..

[182] Olanow C.W., Kieburtz K., Odin P., Espay A.J., Standaert D.G., Fernandez H.H., Vanagunas A., Othman A.A., Widnell K.L., Robieson W.Z. (2014). Double-blind, double-dummy, randomized study of continuous intrajejunal infusion of levodopa-carbidopa intestinal gel in advanced Parkinson’s disease. Lancet Neurol..

[183] Fox S.H., Katzenschlager R., Lim S.Y., Barton B., de Bie R.M.A., Seppi K., Coelho M., Sampaio C., Movement Disorder Society Evidence-Based Medicine Committee (2018). International Parkinson and movement disorder society evidence-based medicine review: Update on treatments for the motor symptoms of Parkinson’s disease. Mov. Disord..

[184] Cacciari B., Spalluto G., Federico S. (2018). A2A Adenosine Receptor Antagonists as Therapeutic Candidates: Are They Still an Interesting Challenge?. Mini Rev. Med. Chem..

[185] (2019). Safinamide for Parkinson’s disease. Aust. Prescr..

[186] Tran T.N., Vo T.N.N., Frei K., Truong D.D. (2018). Levodopa-induced dyskinesia: Clinical features, incidence, and risk factors. J. Neural Transm..

[187] Aquino C.C., Fox S.H. (2015). Clinical spectrum of levodopa-induced complications. Mov. Disord..

[188] Dragašević-Mišković N., Petrović I., Stanković I., Kostić V.S. (2019). Chemical management of levodopa-induced dyskinesia in Parkinson’s disease patients. Expert Opin. Pharmacother..

[189] Vijayakumar D., Jankovic J. (2016). Drug-Induced Dyskinesia, Part 1: Treatment of Levodopa-Induced Dyskinesia. Drugs.

[190] Lin M.M., Laureno R. (2019). Less Pulsatile Levodopa Therapy (6 Doses Daily) Is associated with a Reduced Incidence of Dyskinesia. J. Mov. Disord..

[191] Calabresi P., Di Filippo M., Ghiglieri V., Tambasco N., Picconi B. (2010). Levodopa-induced dyskinesias in patients with Parkinson’s disease: Filling the bench-to-bedside gap. Lancet Neurol..

[192] Sawada H., Oeda T., Kuno S., Nomoto M., Yamamoto K., Yamamoto M., Hisanaga K., Kawamura T., Amantadine Study Group (2010). Amantadine for dyskinesias in Parkinson’s disease: A randomized controlled trial. PLoS ONE.

[193] Del Dotto P., Pavese N., Gambaccini G., Bernardini S., Metman L.V., Chase T.N., Bonuccelli U. (2001). Intravenous amantadine improves levadopa-induced dyskinesias: An acute double-blind placebo-controlled study. Mov. Disord..

[194] Thomas A., Iacono D., Luciano A.L., Armellino K., Di Iorio A., Onofrj M. (2004). Duration of amantadine benefit on dyskinesia of severe Parkinson’s disease. J. Neurol. Neurosurg. Psychiatry.

[195] Dashtipour K., Tafreshi A.R., Pahwa R., Lyons K.E. (2019). Extended-Release Amantadine for Levodopa-Induced Dyskinesia. Expert Rev. Neurother..

[196] Pahwa R., Tanner C.M., Hauser R.A., Isaacson S.H., Nausieda P.A., Truong D.D., Agarwal P., Hull K.L., Lyons K.E., Johnson R. (2017). ADS-5102 (Amantadine) Extended-Release Capsules for Levodopa-Induced Dyskinesia in Parkinson Disease (EASE LID Study): A Randomized Clinical Trial. JAMA Neurol..

[197] Trenkwalder C., Stocchi F., Poewe W., Dronamraju N., Kenney C., Shah A., von Raison F., Graf A. (2016). Mavoglurant in Parkinson’s patients with l-Dopa-induced dyskinesias: Two randomized phase 2 studies. Mov. Disord..

[198] Freitas M.E., Fox S.H. (2016). Nondopaminergic treatments for Parkinson’s disease: Current and future prospects. Neurodegener. Dis. Manag..

[199] Wong K.K., Alty J.E., Goy A.G., Raghav S., Reutens D.C., Kempster P.A. (2011). A randomized, double-blind, placebo-controlled trial of levetiracetam for dyskinesia in Parkinson’s disease. Mov. Disord..

[200] Stathis P., Konitsiotis S., Tagaris G., Peterson D., VALID-PD Study Group (2011). Levetiracetam for the management of levodopa-induced dyskinesias in Parkinson’s disease. Mov. Disord..

[201] Mizuno Y., Kondo T., Japanese Istradefylline Study Group (2013). Adenosine A2A receptor antagonist istradefylline reduces daily OFF time in Parkinson’s disease. Mov. Disord..

[202] Schwarzschild M.A., Agnati L., Fuxe K., Chen J.F., Morelli M. (2006). Targeting adenosine A2A receptors in Parkinson’s disease. Trends Neurosci..

[203] Fernandez H.H., Greeley D.R., Zweig R.M., Wojcieszek J., Mori A., Sussman N.M., 6002-US-051 Study Group (2010). Istradefylline as monotherapy for Parkinson disease: Results of the 6002-US-051 trial. Park. Relat. Disord..

[204] Svenningsson P., Rosenblad C., Af Edholm Arvidsson K., Wictorin K., Keywood C., Shankar B., Lowe D.A., Björklund A., Widner H. (2015). Eltoprazine counteracts l-DOPA-induced dyskinesias in Parkinson’s disease: A dose-finding study. Brain.

[205] Onofrj M., Thomas A. (2005). Acute akinesia in Parkinson disease. Neurology.

[206] Thomas A., Onofrj M. (2005). Akinetic crisis, acute akinesia, neuroleptic malignant-like syndrome, Parkinsonism-hyperpyrexia syndrome, and malignant syndrome are the same entity and are often independent of treatment withdrawal. Mov. Disord..

[207] Martino G., Capasso M., Nasuti M., Bonanni L., Onofrj M., Thomas A. (2015). Dopamine transporter single- photon emission computerized tomography supports diagnosis of akinetic crisis of parkinsonism and of neuroleptic malignant syndrome. Medicine.

[208] Eggers C., Kahraman D., Fink G.R., Schmidt M., Timmermann L. (2011). Akinetic-rigid and tremor-dominant Parkinson’s disease patients show different patterns of FP-CIT single photon emission computed tomography. Mov. Disord..

[209] Danielczyk W. (1995). Twenty-five years of amantadine therapy in Parkinson’s Disease. J. Neural Transm. Suppl..

[210] Stacy M., Factor S. (2004). Rapid treatment of “off” episodes. Will this change Parkinson disease therapy?. Neurology.

[211] Dafotakis M., Sparing R., Juzek A., Block F., Kosinski C.M. (2009). Transdermal dopaminergic stimulation with rotigotine in Parkinsonian akinetic crisis. J. Clin. Neurosci..

[212] Capasso M., De Angelis M.V., Di Muzio A., Anzellotti F., Bonanni L., Thomas A., Onofrj M. (2015). Critical Illness Neuromyopathy Complicating Akinetic Crisis in Parkinsonism: Report of 3 Cases. Medicine.

[213] Elkouzi A., Vedam-Mai V., Eisinger R.S., Okun M.S. (2019). Emerging therapies in Parkinson disease—Repurposed drugs and new approaches. Nat. Rev. Neurol..

[214] Oliver D.J., Borasio G.D., Caraceni A., de Visser M., Grisold W., Lorenzl S., Veronese S., Voltz R. (2016). A consensus review on the development of palliative care for patients with chronic and progressive neurological disease. Eur. J. Neurol..

[215] Van der Marck M.A., Kalf J.G., Sturkenboom I.H., Nijkrake M.J., Munneke M., Bloem B.R. (2009). Multidisciplinary care for patients with Parkinson’s disease. Park. Relat. Disord..

[216] Radder D.L.M., de Vries N.M., Riksen N.P., Diamond S.J., Gross D., Gold D.R., Heesakkers J., Henderson E., Hommel A.L.A.J., Lennaerts H.H. (2019). Multidisciplinary care for people with Parkinson’s disease: The new kids on the block!. Expert Rev. Neurother..

[217] Zigmond M.J., Smeyne R.J. (2014). Exercise: Is it a neuroprotective and if so, how does it work?. Park. Relat. Disord..

[218] Uc E.Y., Doerschug K.C., Magnotta V., Dawson J.D., Thomsen T.R., Kline J.N., Rizzo M., Newman S.R., Mehta S., Grabowski T.J. (2014). Phase I/II randomized trial of aerobic exercise in Parkinson disease in a community setting. Neurology.

[219] Cruickshank T.M., Reyes A.R., Ziman M.R. (2015). A systematic review and meta-analysis of strength training in individuals with multiple sclerosis or Parkinson disease. Medicine.

[220] Shulman L.M., Katzel L.I., Ivey F.M., Sorkin J.D., Favors K., Anderson K.E., Smith B.A., Reich S.G., Weiner W.J., Macko R.F. (2013). Randomized clinical trial of 3 types of physical exercise for patients with Parkinson disease. JAMA Neurol..

[221] Deane K.H., Whurr R., Playford E.D., Ben-Shlomo Y., Clarke C.E. (2001). Speech and language therapy for dysarthria in Parkinson’s disease. Cochrane Database Syst. Rev..

[222] Mahler L.A., Ramig L.O., Fox C. (2015). Evidence-based treatment of voice and speech disorders in Parkinson disease. Curr. Opin. Otolaryngol. Head Neck Surg..

[223] Kalia S.K., Sankar T., Lozano A.M. (2013). Deep brain stimulation for Parkinson’s disease and other movement disorders. Curr. Opin. Neurol..

[224] Deuschl G., Schade-Brittinger C., Krack P., Volkmann J., Schäfer H., Bötzel K., Daniels C., Deutschländer A., Dillmann U., Eisner W. (2006). A randomized trial of deep-brain stimulation for Parkinson’s disease. N. Engl. J. Med..

[225] Follett K.A., Weaver F.M., Stern M., Hur K., Harris C.L., Luo P., Marks W.J., Rothlind J., Sagher O., Moy C. (2010). Pallidal versus subthalamic deep-brain stimulation for Parkinson’s disease. N. Engl. J. Med..

[226] Kalia L.V., Lang A.E. (2015). Parkinson’s disease. Lancet.

[227] Schuepbach W.M.M., Rau J., Knudsen K., Volkmann J., Krack P., Timmermann L., Hälbig T.D., Hesekamp H., Navarro S.M., Meier N. (2013). Neurostimulation for Parkinson’s disease with early motor complications. N. Engl. J. Med..

[228] Grabli D., Karachi C., Folgoas E., Monfort M., Tande D., Clark S., Civelli O., Hirsch E.C., François C. (2013). Gait disorders in parkinsonian monkeys with pedunculopontine nucleus lesions: A tale of two systems. J. Neurosci..

[229] Jenkinson N., Nandi D., Miall R.C., Stein J.F., Aziz T.Z. (2004). Pedunculopontine nucleus stimulation improves akinesia in a Parkinsonian monkey. Neuroreport.

[230] Weiss D., Walach M., Meisner C., Fritz M., Scholten M., Breit S., Plewnia C., Bender B., Gharabaghi A., Wächter T. (2013). Nigral stimulation for resistant axial motor impairment in Parkinson’s disease? A randomized controlled trial. Brain.

[231] Gilat M., Lígia Silva de Lima A., Bloem B.R., Shine J.M., Nonnekes J., Lewis S.J.G. (2018). Freezing of gait: Promising avenues for future treatment. Park. Relat. Disord..

[232] Kim M.S., Chang W.H., Cho J.W., Youn J., Kim Y.K., Kim S.W., Kim Y.H. (2015). Efficacy of cumulative high-frequency rTMS on freezing of gait in Parkinson’s disease. Restor. Neurol. Neurosci..

[233] Broeder S., Nackaerts E., Heremans E., Vervoort G., Meesen R., Verheyden G., Nieuwboer A. (2015). Transcranial direct current stimulation in Parkinson’s disease: Neurophysiological mechanisms and behavioral effects. Neurosci. Biobehav. Rev..

[234] Valentino F., Cosentino G., Brighina F., Pozzi N.G., Sandrini G., Fierro B., Savettieri G., D’Amelio M., Pacchetti C. (2014). Transcranial direct current stimulation for treatment of freezing of gait: A cross-over study. Mov. Disord..

[235] Dagan M., Herman T., Harrison R. (2018). Multitarget transcranial direct current stimulation for freezing of gait in Parkinson’s disease. Mov. Disord..

[236] Dobkin R.D., Menza M., Allen L.A., Gara M.A., Mark M.H., Tiu J., Bienfait K.L., Friedman J. (2011). Cognitive-behavioral therapy for depression in Parkinson’s disease: A randomized, controlled trial. Am. J. Psychiatry.

[237] Chen J., Marsh L. (2014). Anxiety in Parkinson’s disease: Identification and management. Adv. Neurol. Disord..

[238] Folkerts A.K., Dorn M.E., Roheger M., Maassen M., Koerts J., Tucha O., Altgassen M., Sack A.T., Smit D., Haarmann L. (2018). Cognitive Stimulation for Individuals with Parkinson’s Disease Dementia Living in Long-Term Care: Preliminary Data from a Randomized Crossover Pilot Study. Park. Dis..

[239] McCormick S.A., Vatter S., Carter L.A., Smith S.J., Orgeta V., Poliakoff E., Silverdale M.A., Raw J., Ahearn D.J., Taylor C. (2019). Parkinson’s-adapted cognitive stimulation therapy: Feasibility and acceptability in Lewy body spectrum disorders. J. Neurol..

[240] Ferreira J.J., Katzenschlager R., Bloem B.R., Bonuccelli U., Burn D., Deuschl G., Dietrichs E., Fabbrini G., Friedman A., Kanovsky P. (2013). Summary of the recommendations of the EFNS/MDS-ES reviewon therapeutic management of Parkinson’s disease. Eur. J. Neurol..

[241] Fasano A., Daniele A., Albanese A. (2012). Treatment of motor and non-motor features of Parkinson’s disease with deep brain stimulation. Lancet Neurol..

[242] Biundo R., Weis L., Fiorenzato E., Gentile G., Giglio M., Schifano R., Campo M.C., Marcon V., Martinez-Martin P., Bisiacchi P. (2015). Double-blind Randomized Trial of tDCS Versus Sham in Parkinson Patients with Mild Cognitive Impairment Receiving Cognitive Training. Brain Stimul..

[243] Elsner B., Kugler J., Pohl M., Mehrholz J. (2016). Transcranial direct current stimulation (tDCS) for idiopathic Parkinson’s disease. Cochrane Database Syst. Rev..

[244] Park A., Stacy M. (2015). Disease-Modifying Drugs in Parkinson’s Disease. Drugs.

[245] Lang A.E., Espay A.J. (2018). Disease Modification in Parkinson’s Disease: Current Approaches, Challenges, and Future Considerations. Mov. Disord..

[246] Beal M.F., Oakes D., Shoulson I., Henchcliffe C., Galpern W.R., Haas R., Juncos J.L., Nutt J.G., Voss T.S., Ravina B. (2014). A randomized clinical trial of high-dosage coenzyme Q10 in early Parkinson disease: No evidence of benefit. JAMA Neurol..

[247] Kieburtz K., Tilley B.C., Elm J.J., Babcock D., Hauser R., Ross G.W., Augustine A.H., Augustine E.U., Aminoff M.J., Writing Group for the NINDS Exploratory Trials in Parkinson Disease (NET-PD) Investigators (2015). Effect of creatine monohydrate on clinical progression in patients with Parkinson disease: A randomized clinical trial. JAMA.

[248] Schenk D.B., Koller M., Ness D.K., Griffith S.G., Grundman M., Zago W., Soto J., Atiee G., Ostrowitzki S., Kinney G.G. (2017). First-in-human assessment of PRX002, an anti-alpha-synuclein monoclonal antibody, in healthy volunteers. Mov. Disord..

[249] Karuppagounder S.S., Brahmachari S., Lee Y., Dawson V.L., Dawson V.L., Dawson T.M., Ko H.S. (2014). The c-Abl inhibitor, nilotinib, protects dopaminergic neurons in a preclinical animal model of Parkinson’s disease. Sci. Rep..

[250] McNeill A., Magalhaes J., Shen C., Chau K.Y., Hughes D., Mehta A., Foltynie T., Cooper J.M., Abramov A.Y., Gegg M. (2014). Ambroxol improves lyso- somal biochemistry in glucocerebrosidase mutation-linked Parkin-son disease cells. Brain.

[251] Zhao H.T., John N., Delic V., Ikeda-Lee K., Kim A., Weihofen A., Swayze E.E., Kordasiewicz H.B., West A.B., Volpicelli-Daley L.A. (2017). LRRK2 Antisense Oligonucleotides Ameliorate α-Synuclein Inclusion Formation in a Parkinson’s Disease Mouse Model. Mol. Ther. Nucleic Acids.

[252] Surmeier D.J., Obeso J.A., Halliday G.M. (2017). Selective neuronal vulnera-bility in Parkinson disease. Nat. Rev. Neurosci..

[253] Simuni T. A phase 3 study of isradipine as a disease-modifying agent in patients with early Parkinson’s disease (STEADY-PD III): Final study results. Proceedings of the 2019 American Academy of Neurology Annual Meeting.

[254] Mann V.M., Cooper J.M., Daniel S.E., Srai K., Jenner P., Marsden C.D., Schapira A.H. (1994). Complex I, iron, and ferritin in Parkinson’s disease substantia nigra. Ann. Neurol..

[255] Athauda D., Maclagan K., Skene S.S., Bajwa-Joseph M., Letchford D., Chowdhury K., Hibbert S., Budnik N., Zampedri L., Dickson J. (2017). Exenatide once weekly versus placebo in Parkinson’s disease: A randomised, double-blind, placebo-controlled trial. Lancet.

[256] Athauda D., Foltynie T. (2016). The glucagon-like peptide 1 (GLP) recep-tor as a therapeutic target in Parkinson’s disease: Mechanisms of action. Drug Discov. Today.

[257] Postuma R.B., Anang J., Pelletier A., Joseph L., Moscovich M., Grimes D., Furtado S., Munhoz R.P., Appel-Cresswell S., Moro A. (2017). Caffeine as symptomatic treatment for Parkinson disease (Cafe-PD): A randomized trial. Neurology.

[258] Carroll C.B., Wyse R.K.H. (2017). Simvastatin as a Potential Disease-Modifying Therapy for Patients with Parkinson’s Disease: Rationale for Clinical Trial, and Current Progress. J. Park. Dis..

[259] Quik M., Parameswaran N., McCallum S.E., Bordia T., Bao S., McCormack A., Kim A., Tyndale R.F., Langston J.W., Di Monte D.A. (2006). Chronic oral nicotine treatment protects against striatal degeneration in MPTP-treated primates. J. Neurochem..

[260] Mak M.K., Wong-Yu I.S., Shen X., Chung C.L. (2017). Long-term effects of exercise and physical therapy in people with Parkinson disease. Nat. Rev. Neurol..

